# Long-term solar radiation forecasting in India using EMD, EEMD, and advanced machine learning algorithms

**DOI:** 10.1007/s10661-025-13738-8

**Published:** 2025-02-18

**Authors:** T. RajasundrapandiyanLeebanon, N. S. Sakthivel Murugan, K. Kumaresan, Andrew Jeyabose

**Affiliations:** 1 Department of Electrical and Electronics Engineering, TamilNadu College of Engineering, Coimbatore, Tamil Nadu India; 2 Department of Mechanical Engineering, Park College of Engineering & Technology, Coimbatore, TamilNadu India; 3 Department of Computer Science and Engineering, Manipal Institute of Technology, Manipal Academy of Higher Education, Manipal, Karnataka 576104 India

**Keywords:** Long-term solar radiation, Machine learning, Empirical mode decomposition, Ensemble empirical mode decomposition, Indian cities

## Abstract

Solar radiation plays a critical role in the carbon sequestration processes of terrestrial ecosystems, making it a key factor in environmental sustainability among various renewable energy sources. This study integrates two advanced signal processing techniques—empirical mode decomposition (EMD) and ensemble empirical mode decomposition (EEMD)—with machine learning (ML) algorithms, including multilayer perceptron (MLP), random forest regression (RFR), support vector regression (SVR), and ridge regression, to forecast long-term solar radiation. Meteorological data spanning 13 years (2000–2012) from seven locations across India (Bhubaneswar, Chennai, Delhi, Hyderabad, Nagpur, Patna, and Trivandrum) were used for training and testing. The optimal model was identified based on performance metrics, including the highest linear correlation coefficient (*R*), and the lowest mean absolute error (MAE) and root mean square error (RMSE). The results indicate that EEMD integrated with ML algorithms consistently outperformed EMD-based approaches. Among the ML models evaluated, EEMD integrated with MLP achieved the best performance across all locations, with RMSE = 0.332, MAE = 0.26, and *R*^2^ = 0.99. Furthermore, a comparative analysis with previous studies demonstrated that the proposed approach provides superior accuracy, underscoring its efficacy in solar radiation forecasting.

## Introduction

Renewable energy sources, especially solar, geothermal, and wind energy are acknowledged for their environmental benefits. Among these, solar energy stands for its accessibility, easy availability and its non-polluting characteristics for its nonemission of harmful gases such as carbon dioxide (CO^2^) by Singh et al. (2012). Solar energy plays a vital role in energy security and sustainability (Alanazi et al., 2016). It is the primary source of energy in the atmosphere, accounting for more than 99.97% of all untapped energy (Blal et al., 2020). The sun’s energy received as electromagnetic radiations is further converted into power (Premalatha & Valan Arasu, 2016). The measuring instruments such as pyranometers and pyrheliometers are used to monitor the daily global solar radiation (GSR). However, this method is too expensive and time consuming. Solar forecasting is considered as an efficient alternative for effective energy usage. Approxiamately four million exajoules (1 EJ = 1018 J) of solar energy reach the earth’s surface in which 5 × 104 EJ is claimed to be harvested (Kabir et al., 2018). Energy imbalances can be tackled with solar forecasting by reducing it to 19.65% (Kaur et al., 2016). In recent days, solar energy has attracted many researchers which leads to the fast development of long-term solar forecasting methods.

### Statistical methods for solar forecasting

Short-term solar forecasting methods are divided into main categories: traditional statistical methods and artificial intelligence methods (Jebaraj & Iniyan, 2006; Yin et al., 2019). Traditional methods consists of dynamic approach and empirical model. Based on the sunshine duration, the dynamic approaches predicts short-term radiation while empirical approaches estimate GSR which leads to achieve high predictive accuracy by Mohanty et al. (2016). Notable statistical models includes ARIMA (autoregressive integrated moving average) models, Bayesian estimation models, and Kalman filtering models, in which the ARIMA algorithm captures temporal structures in time series data and are suitable for linear temporal patterns but not suitable for nonlinear temporal patterns in real world scenarios by Siami-Namini et al. (2018); the Angstrom–Prescott sunshine–based empirical model is a widely used approach for estimating global solar radiation with the performance metrics of mean bias error (MBE), mean percentage error (MPE), and root mean square error (RMSE) (Mohanty et al., 2016; Ronno et al., 2017). Due to various climatic conditions, empirical models needs long processing time for the prediction of GSR (Perveen et al., 2018). Based on the recent studies, empirical models and hybrid support vector machine are less efficient compared to machine learning techniques (Fan et al., 2019, 2020).

### Machine learning for solar forecasting

Machine learning models have been applied in this field of solar radiation forecasting to capture complex relationships. Artificial neural network (ANN) is an efficient ML-based model for solar forecasting. For example Meenal and Selvakumar (2018) utilized seven different Indian cities, integrates multiple empirical models, in which, artificial neural networks (ANN) achieved good results. Srivastava et al. (2019) compared four ML techniques such as multivariate adaptive regression splines (MARS), M5 (decision tree machine learning methods), classification and regression tree (CART), and random forest (RF) used for solar radiation forecasting, concluding that RF model provided best results for projecting solar radiation. Maldonado et al. (2019) developed a strategic framework for automated selection of lag in time series analysis with support vector regression (SVR). This strategy was applied on four datasets for short-term predictions of solar forecasting. However, the computational cost of the algorithms is observed to increase exponentially with the number of preselected variables. Vennila et al. (2022) suggest that machine learning techniques, particularly hybrid models and artificial neural networks, are effective for improving the accuracy of solar energy production forecasting by accounting for weather variability and optimizing model performance. Anuradha et al. (2021) investigated random forest regressor (RFR), SVM regressor, and linear regressor for the solar power–based generation forecast. The RFR yielded the best results with accuracy of 94.01, RMSE 27.32, MAE 12.45, and MSE 746.48 respectively. Hedar et al. (2021) investigated the GPR technique for the prediction of solar-based radiation. The proposed model gave an RMSE of 421.15 W/m^2^ respectively. Mohanad et al. (2018) adopted six daily climate variables for the eleven major locations, and optimal data driven technique based on SVR model using PSO algorithm. The PSO-SVR model outperformed MARS and SVR. Zhang and Hong (2019) support energy policy and managers of power systems, a new forecasting model, the CEEMDAN- SVRQDA model is proposed to provide more accurate forecasts. Ghimire et al. (2023) developed an electricity forecast model by using multi-head self-attention transformer. This model achieves high accuracy and low predictive errors. The main objective of this work is to improve power demand point prediction. Kundra and Sadawarti (2015) introduce a novel approach of hybridization of cuckoo search and particle swarm optimization for remote sensing image classification. This model achieves an accuracy of 96.33%. Deo et al. (2022) present an artificial intelligence (AI) approach that incorporates total sky conditions, focusing on the impact of cloud cover variations to accurately model PPFD at 5-min timescales. The novelty and contribution lie in developing the first deep learning AI method for real-time PPFD forecasting, effectively capturing the influence of cloud properties on measured photosynthetically active radiation. Dong et al. (2018) proposed seasonal SVR with CCS, called the support vector regression (SVR) with chaotic cuckoo search (SSVRCCS) model, which is designed to enhance forecasting accuracy by effectively capturing the non-linear and cyclical patterns of electric load variations. This model achieves more high forecasting accurate levels. Almarzooqi et al. (2024) proposed hybrid framework which employs a fast trainable statistical learning technique based on the truncated-regularized kernel ridge regression model for optimal prediction of grid-connected solar photovoltaic (PV) power plants. Ahmed et al. (2024) develop a hybrid convolutional neural network long short-term memory bidirectional gated recurrent unit forecast system (CLSTM-BIGRU) trained to accurately predict significant wave height at multiple forecasting horizons. Hong et al. (2019) present hybrid kernel–based SVR to stimulate the motion of a floating platform with EEMD to forecast the motion data with reliable accuracy and effectiveness with a chaotic efficient bat algorithm to receive an optimized parameter.

### Literature-based research gaps and motivation for the proposed model

The application of machine learning in forecasting solar radiation holds significant promise; however, still uncertainties remain constant in developing models and methodologies (Sivakumar et al., 2023). The primary cause of resulting randomness in predicted values caused by the inherent variability of solar radiation, which is amplified by environmental factors such as cloud cover, temperature, and other local weather conditions and air quality such as particulate matter and dust, latitude, and season. Understanding these complexities is crucial for enhancing predictive accuracy. To address these challenges, a comprehensive literature analysis has been conducted, followed by an exploration of existing research gaps. Wang et al. (2022) demonstrated the comparison between single, stand-alone models to hybrid models for solar radiation prediction. According to their findings, single models offer lack of accuracy which indicates a clear need for advancement in modeling strategies and the development of hybrid techniques. To increase the forecasting accuracy of the ML and deep learning (DL) models, the feasible hybrid option is to utilize signal processing techniques to remove noise from data thereby enhancing the signal clarity and extraction of relevant features required to improve the prediction accuracy.

For instance, AL‐Musaylh et al. (2021) introduce a novel empirical wavelet transform (EWT) technique to analyze daily gas consumption demand patterns in Melbourne, Australia, and forecast future demand using a hybrid decision tree (M5 model tree) model. The EWT algorithm decomposes the data into intrinsic mode functions (IMFs), capturing frequency patterns and stochastic behaviors, and significantly improves forecasting accuracy, with the EWT-M5 model tree outperforming traditional methods by achieving a lower RRMSE of 29.19%. Aghmadi et al. (2021) have combined EMD technique with back propagation in neural networks (BPNN) technique for a hybrid-based solar forecasting. The model has produced a root mean square error (RMSE) of 28.13 W/m^2^. Li et al. (2018) have proposed a hybrid EMD-ANN–based model. The model has yielded an optimal correlation coefficient of 0.93 in terms of monthly predicted values. To anticipate intra-hour solar photovoltaic energy for Vitoria-Gasteiz, Spain, Rodríguez et al. (2022) have suggested a hybrid technique employing Daubechies wavelets and fast Fourier neural network (FFNN). During validation, the hybrid technique has attained an RMSE of 35.7 W/m^2^. In order to perform solar forecasts for Odisha, India, Majumder et al. (2018) presented variational mode decomposition (VMD) with extreme learning machine technique. For a 15-min timeframe forecast, the proposed methodology has produced RMSE and mean absolute percentage error (MAPE) values of 0.011 and 1.244, respectively. An innovative hybrid model for electricity demand forecasting is proposed, integrating the VMD method, self-recurrent mechanism, tent mapping function, out-bound-back mechanism, CS algorithm, and SVR model, referred to as the VMD-SR-SVRCBCS model. The performance of the model receives the significance under 95 confident levels (Zhang & Hong, 2019). Similar to this, there are a few research on ML and hybrid models in the literature for predicting solar radiation; the authors, Li et al. (2018), Sivakumar et al. (2023), Mohanty et al. (2016), Shariff and Duzan (2018), and Mohammadi et al. (2015), have conducted solar forecasting for Indian regions. Majumder et al. (2018) and Maldonado et al. (2019) have demonstrated the integration of machine learning (ML) methods with various signal processing methods like EMD, VMD, and wavelet transforms. Despite of this advancement, the potentials of ensemble empirical mode decomposition (EEMD) algorithm remain unexplored in the field of solar forecasting. Based on this, the following research gaps are explored.

- This paper presents a novel hybrid model that effectively addresses non-stationarity issues in multiple predictor inputs through a self-adaptive approach, while generating accurate forecasts of long-term solar radiation. This model demonstrates enhanced potential for practical applications.
- To achieve this goal, EEMD addresses the mode mixing problem and improves robustness.
- The algorithms such as MLP, SVM, KNN, and Ridge are becoming more prominent due to recent advancements in AI; however, their integration with EEMD and other signal-processing approaches for solar forecasting applications yield significant improvement in its performance.

### Key contributions of the research work

1. The proposed methods focus on solar radiation forecasting with relevance of signal processing techniques such as empirical mode decomposition (EMD) and ensemble empirical mode decomposition (EEMD) with four unique ML algorithms.
2. Effectively decompose non-linear and non-stationary solar data using signal processing techniques such as EMD and EEMD based on temporal-spatial distribution and its computational complexity in terms of series of intrinsic mode functions (IMFs).
3. Four ML techniques are applied to the extracted features to show their efficacy in performance in terms of regression.
4. The multi-layer perceptron (MLP) with EEMD has given better performance compared with EMD in terms of MAE, RMSE, and correlation coefficient.

## Solar radiation forecasting frameworks and evaluation metrics

To achieve the objectives of this research work, the Indian regions has been identified from the relevant datasets. The selection of locations and datasets are based on specific criteria, including geographic diversity and the availability of data. Well suitable framework is designed for the implementation of proposed model. To evaluate the effectiveness of the proposed algorithm, established performance metrics, including mean absolute error (MAE), root mean squared error (RMSE), and *R* squared (*R*^2^) have been identified.

### Data regions and data acquisition

The research work focus on seven major cities known for its significant solar potential. The location includes Bhubaneswar, Chennai, Delhi, Hyderabad, Nagpur, Patna, and Trivandrum. The Indian Meteorological Department (IMD) in Pune provides the input datasets needed for the analysis (https://imdpune.gov.in/). Table 1 lists the geographic information for the seven cities, including longitude, latitude, and the time period during which data was collected. Due to the inability to collect the most recent data for all cities within the same timeframe, outdated datasets with varying timeframes were utilized. To ensure better comparison of the hybrid models, homogeneous parameters were selected, despite the differences in time periods among the chosen cities. The parameters considered include bright sunlight hours (BHSS), day length, and minimum (*T*min) and maximum (*T*max) temperatures.

**Table 1:** Geographical parameters for seven locations

Cities	Latitude	Longitude	Time period	Climatic classification
Bhubaneswar	20.296	85.824	2003–2008	Savanna tropical
Chennai	13.082	80.271	2003–2011	Savanna tropical
Delhi	28.704	77.102	2003–2011	Monsoon-influenced humid subtropical and semi-arid
Hyderabad	17.385	78.486	2000–2008	Tropical wet and dry
Nagpur	21.145	79.088	2004–2010	Savanna tropical
Patna	25.594	85.137	2000–2008	Humid subtropical
Trivandrum	8.524	76.936	2005–2012	Savanna tropical

### Solar forecasting–based framework

**Fig. 1:**
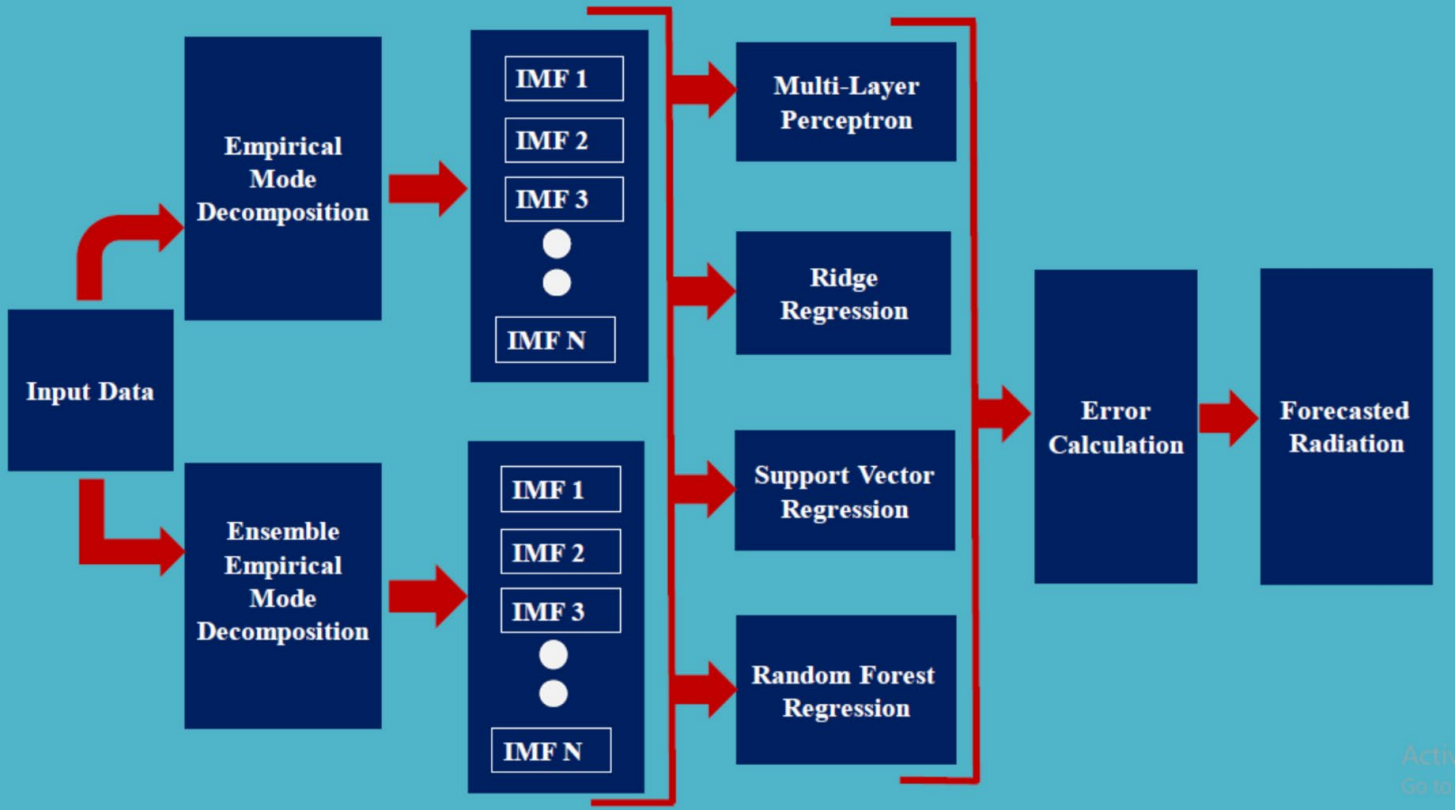
Signal processing-incorporated machine learning techniques for solar radiation forecasting framework

Forecasting-related research typically involves a large datasets, as noted in the “Data regions and data acquisition” section. The datasets contains a wide range of attributes and values, which leads to the improvement of data quality. Various traits and values in the dataset lead to the effective analysis of the framework. Figure 1 shows the systematic architectural diagram for solar radiation forecasting. To improve the quality of the dataset, the noisy values were removed and missing values were addresses using mean imputation. After the pre-processing phase, significant features such as BHSS, *T*min, and *T*max were identified. The signal processing methods such as EMD and EEMD were applied to the pre-processed dataset to generate decomposed values as IMF. Lastly, the ML algorithms are loaded with these data as inputs to calculate the RMSE and MAE, respectively.

### Evaluation metrics for validating model performance

The performance metrics namely, RMSE, MAE, and *R*, are utilized for this study to access the result obtained using each model. RMSE represents degree in dispersion which results in various patterns. For the best accuracy, values of RMSE and MAE should be adjacent to zero while *R* value should be nearer to 1. Equations (1), (2), and (3) describe the equations to compute RMSE, MAE, and *R*^2^ values, respectively by Hedar et al. (2021).

1 $$\text{Root mean square error }(\text{RMSE})=\sqrt{\frac{1}{n}} \sum_{k=1}^{n}{({X}_{\text{actual}}\left(i\right)-{X}_{\text{predicted}}\left(i\right))}^{2}$$

2 $$\text{Mean absolute error }(\text{MAE})=\frac{1}{n}\sum_{k=1}^{n}{\left|\left.{X}_{\text{actual}}\left(i\right)-{X}_{\text{predicted}}\left(i\right)\right|\right.}^{2}$$

3 $$\text{Correlation coefficient }\left(R\right)= \frac{\sum ({X}_{i-}{X}_{\text{mean}})({Y}_{i-}{Y}_{\text{mean}})}{\sqrt{\sum {({X}_{i-}{X}_{\text{mean}})}^{2}{({Y}_{i-}{Y}_{\text{mean}})}^{2}}}$$

## Methodologies

The dataset utilized in this research comprises data from seven cities in India: Bhubaneswar, Chennai, Delhi, Hyderabad, Nagpur, Patna, and Trivandrum. The features considered include BHSS, minimum temperature (*T*_min), and maximum temperature (*T*_max). To enhance the efficacy of the proposed analysis, constant features such as latitude, longitude, and day length were excluded. The dataset was partitioned into training and test sets in a 75–25 ratio to ensure robust evaluation and validation of the classification models. This partitioning facilitates adequate data availability for model training while preserving a significant subset for testing.

Empirical mode decomposition (EMD) is employed to decompose the signal into intrinsic mode functions (IMFs) based on local extrema. Each IMF must adhere to specific properties, including zero mean and symmetry. Ensemble empirical mode decomposition (EEMD) augments the original signal with white noise to mitigate mode mixing and enhance robustness. Once the IMFs reach a normalized level, the features extracted from these IMFs are utilized as input for four distinct regression models for solar radiation forecasting: multi-layer perceptron (MLP) regression, random forest regression (RFR), ridge regression, and support vector regression (SVR).

### Signal processing techniques

#### Empirical mode decomposition

EMD is a flexible method of generating an infinite set of IMF components to represent the source data. The EMD process receives instant frequency data from non-stationary and non-linear data which allows splitting each complex dataset into several limited and often small components. Empirical methods are considered for processing decomposition signals to create inputs for MLP. Each signal is classified as four or five IMFs to generate a series of signals and can reflect the specific physical meaning of the original signal. The IMFs should satisfy the subsequent conditions:

- The number of endpoints and zero crossings must be equal or differ by a maximum of one during the whole duration of a single IMF.
- For each data location, the average envelope determined by the neighborhood maximum and envelope determined by the local maximum is zero.

The original signal can be represented using Eq. (4).

4 $$y\left(x\right)=\sum_{x}{y}_{x}\left(a\right)+R(a)$$

The steps carried out in EMD could be described in an algorithmic form is as follows.

**Algorithm 1:**
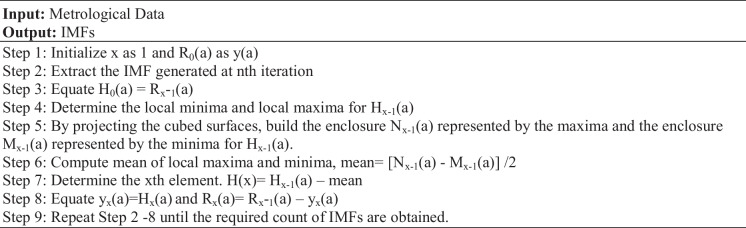
EMD algorithm

**Fig. 2:**
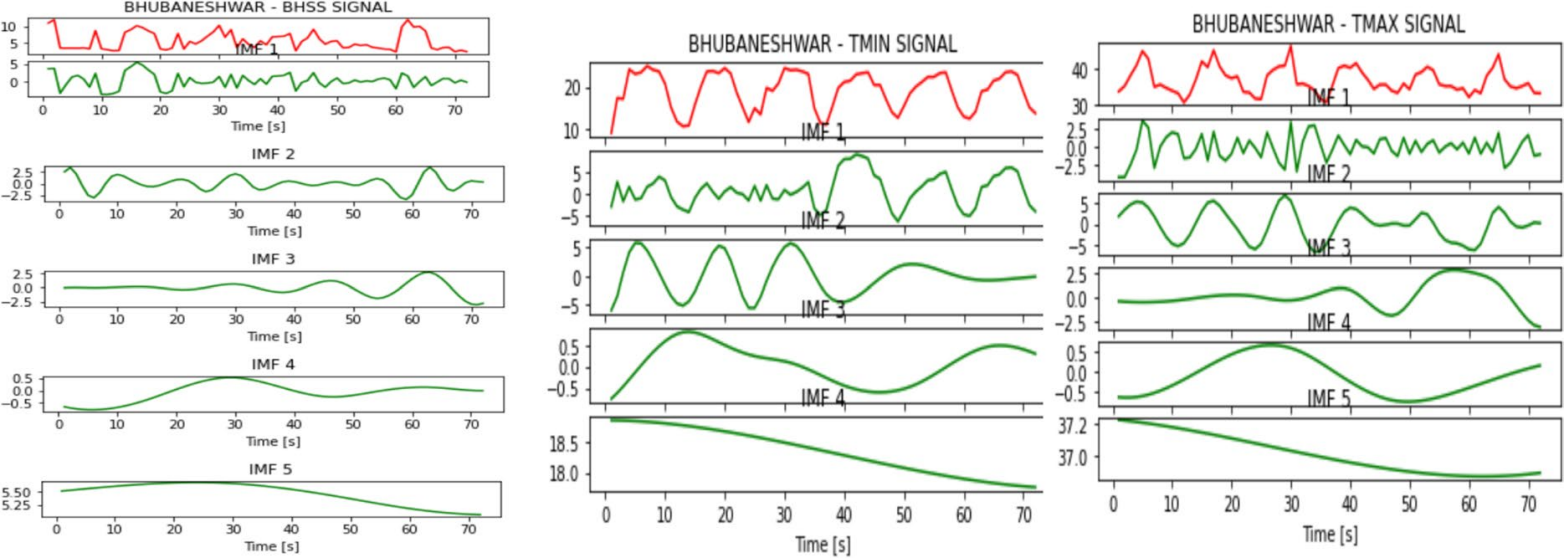
IMFs generated for Bhubaneshwar using EMD

**Fig. 3:**
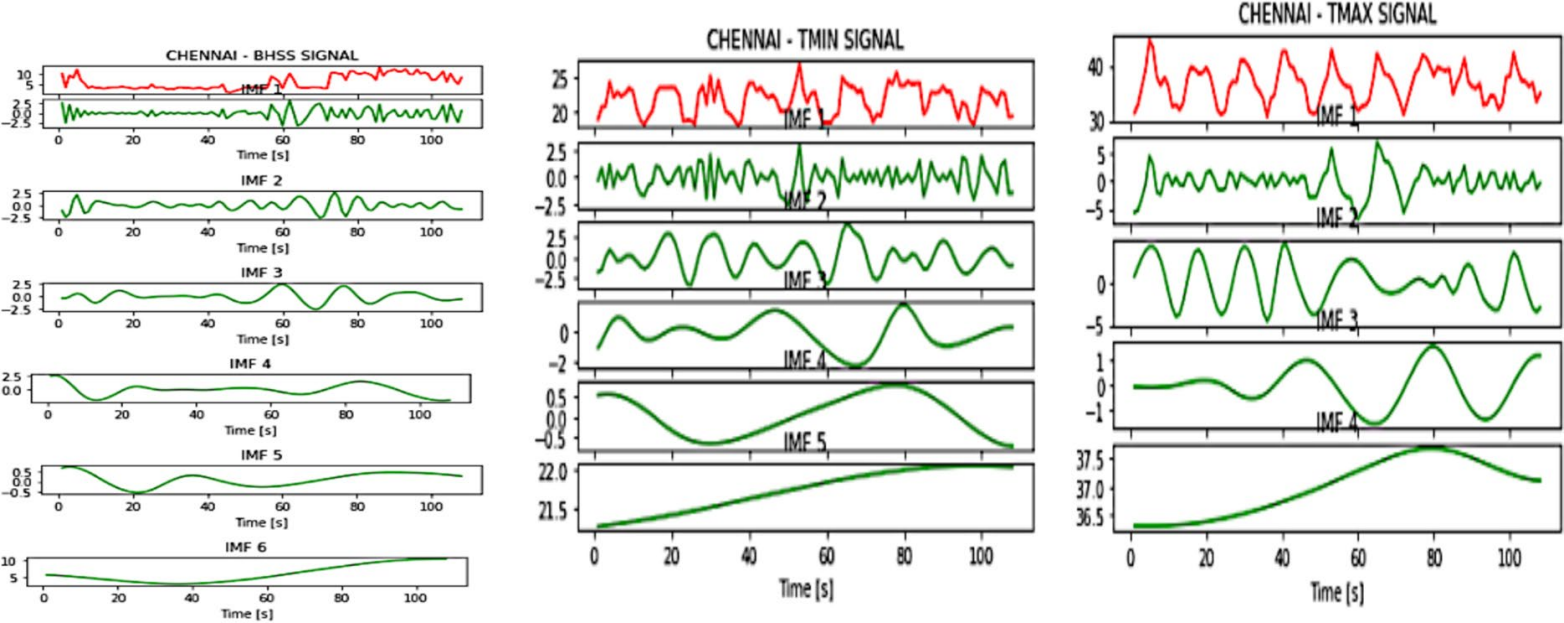
IMFs generated for Chennai using EMD

**Fig. 4:**
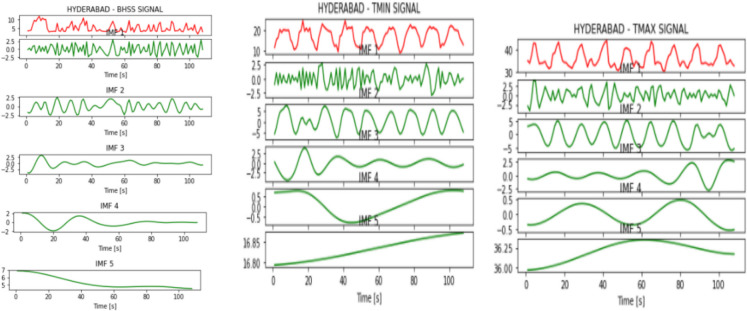
IMFs generated for Hyderabad using EMD

**Fig. 5:**
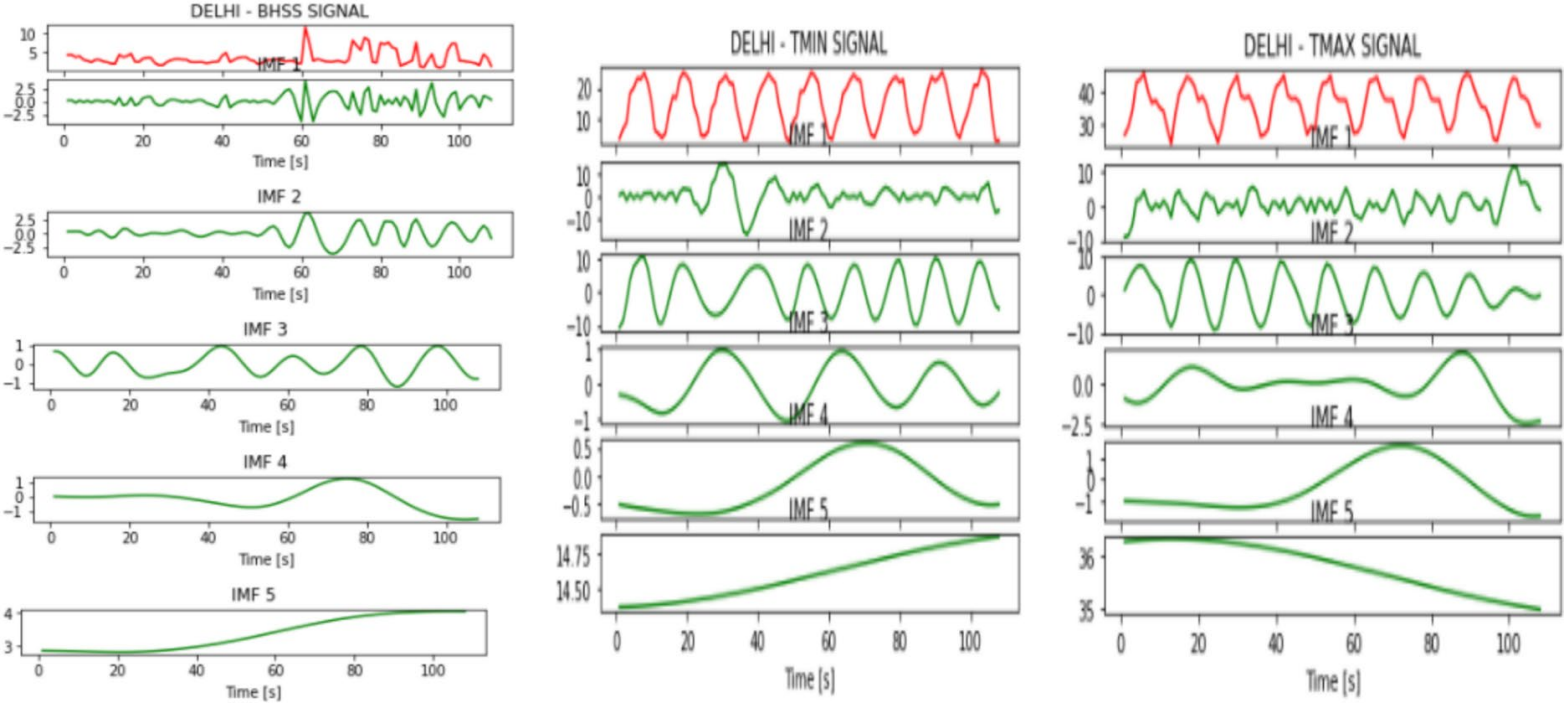
IMFs generated for Delhi using EMD

**Fig. 6:**
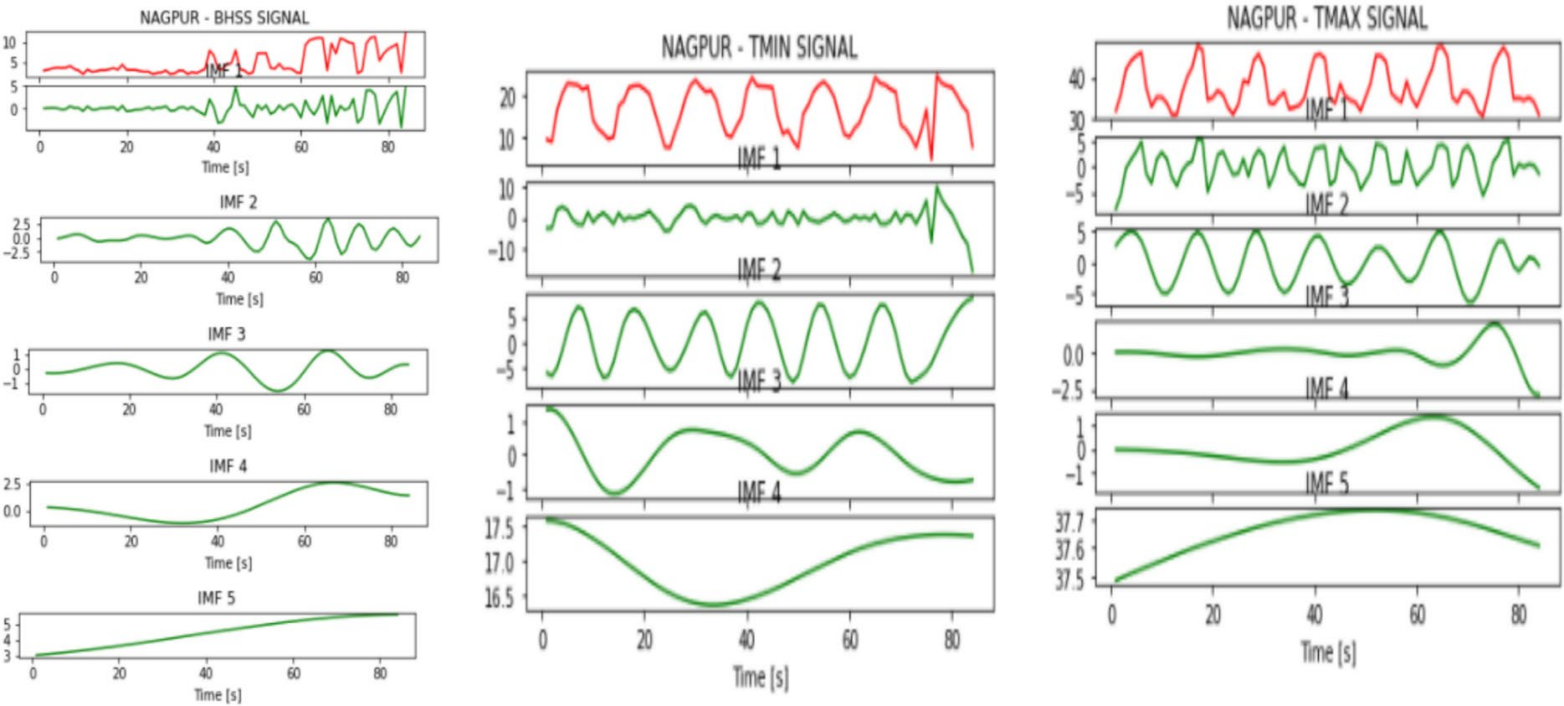
IMFs generated for Nagpur using EMD

**Fig. 7:**
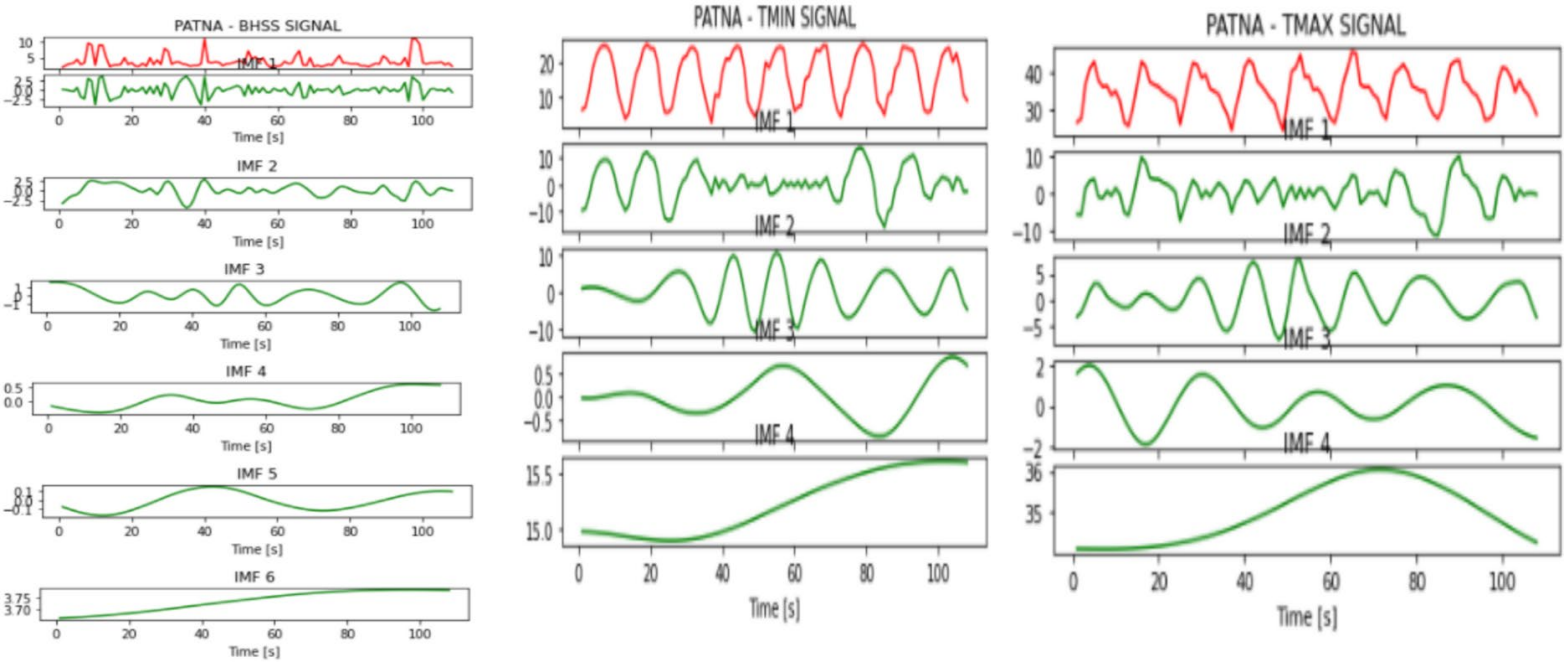
IMFs generated for Patna using EMD

**Fig. 8:**
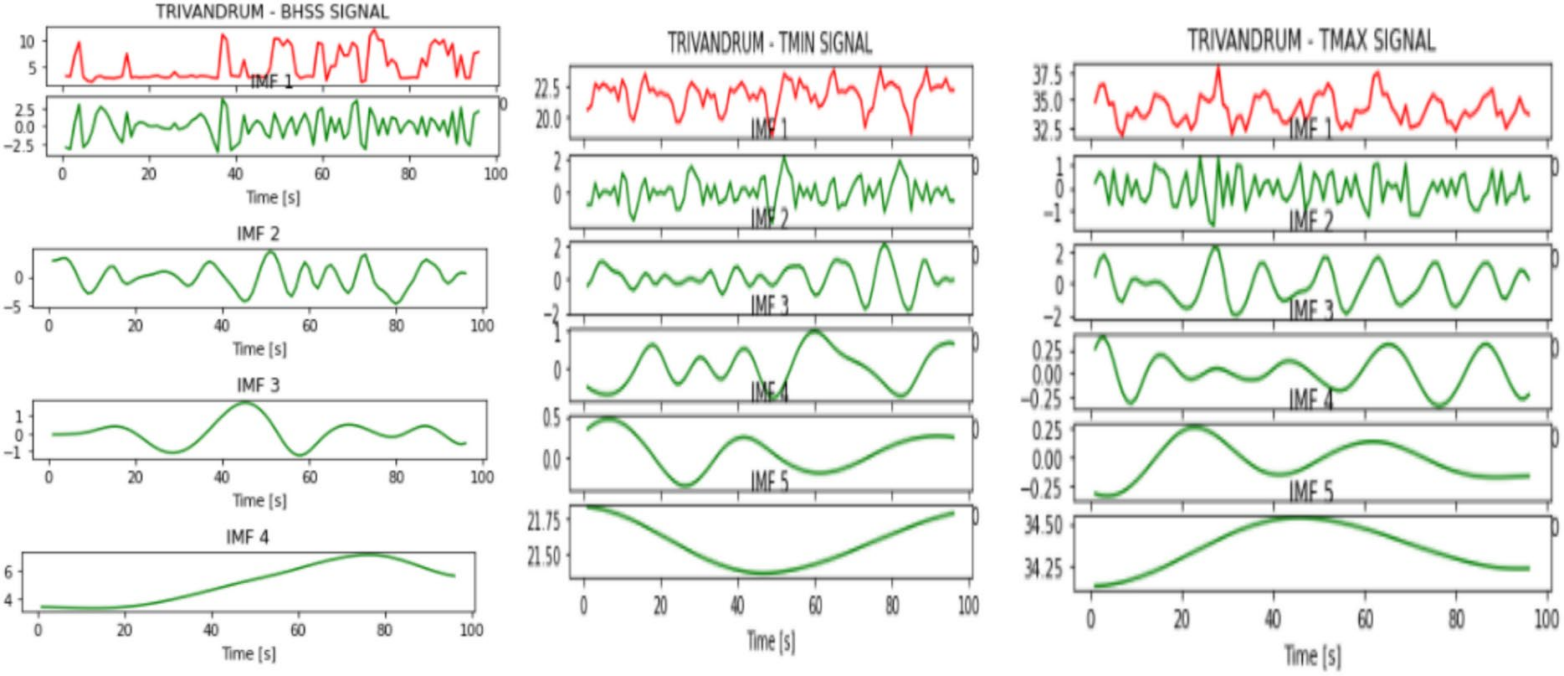
IMFs generated for Trivandrum using EMD

Figures 2, 3, 4, 5, 6, 7, and 8 represent the IMFs generated for the seven states we have considered in this study by the EMD technique.

#### Ensemble empirical mode decomposition

The effect of modes merging is the fundamental downside of EMD. This happens when vibrations with different time spans are stored in one IMF or vibrations with the same time scale are sifted into different IMFs. Wu and Huang (2009) suggested a leading version of the EMD that solves this difficulty. It marks a significant advancement in the EMD approaches (Wu & Huang, 2009). Process of the EEMD is developed as follows:

**Algorithm 1:**
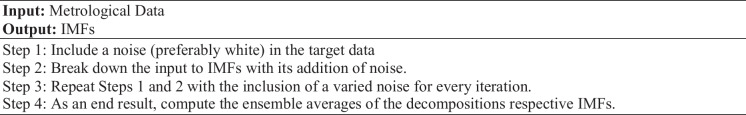
EEMD algorithm

Equation (5) represents the final outcome acquired after the EEMD process. Here, an ensemble of data sets *y*_*i*_(*a*) is created by adding white noise *x*_*ik*_(*a*) to the original time series *y*(*a*).

5 $${y}_{i}\left(a\right)=\frac{1}{t}{\sum }_{k-1}^{t}{x}_{ik}\left(a\right)$$

**Fig. 9:**
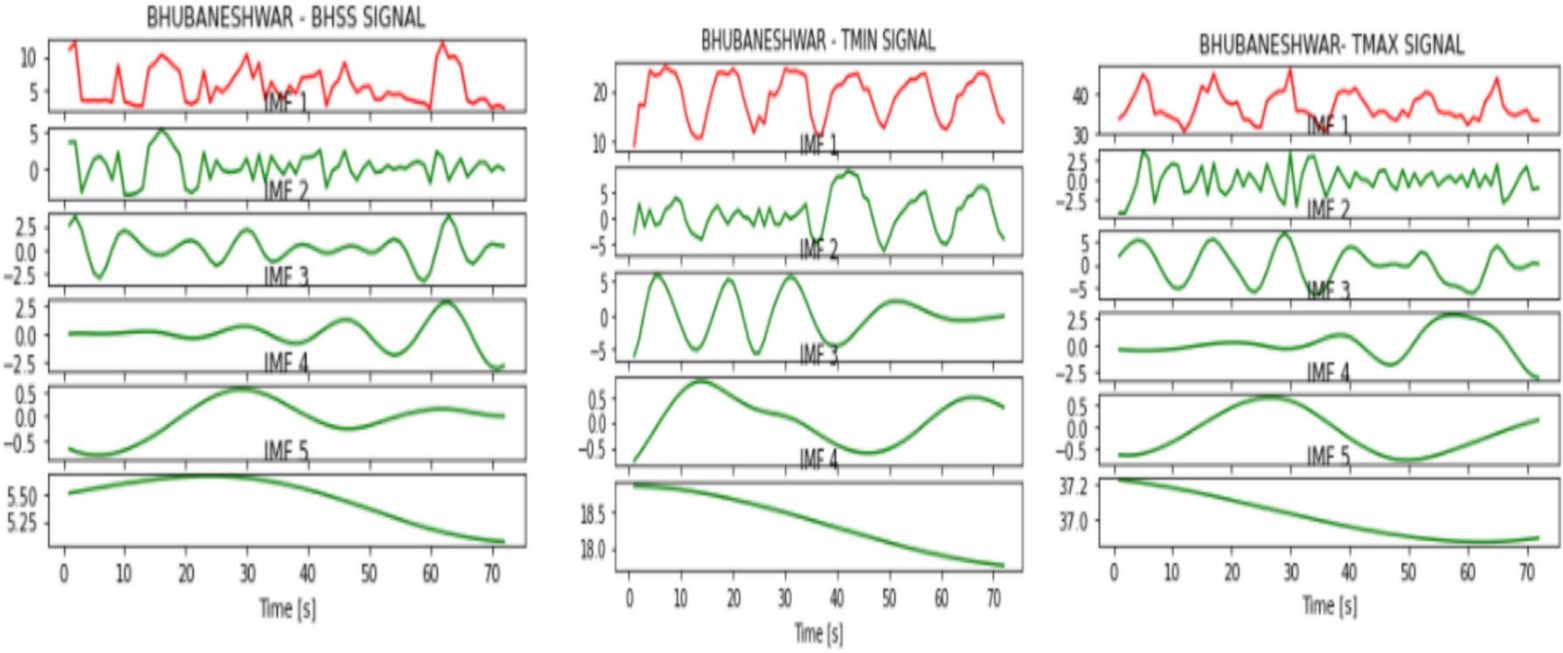
IMFs generated for Bhubaneshwar using EEMD

**Fig. 10:**
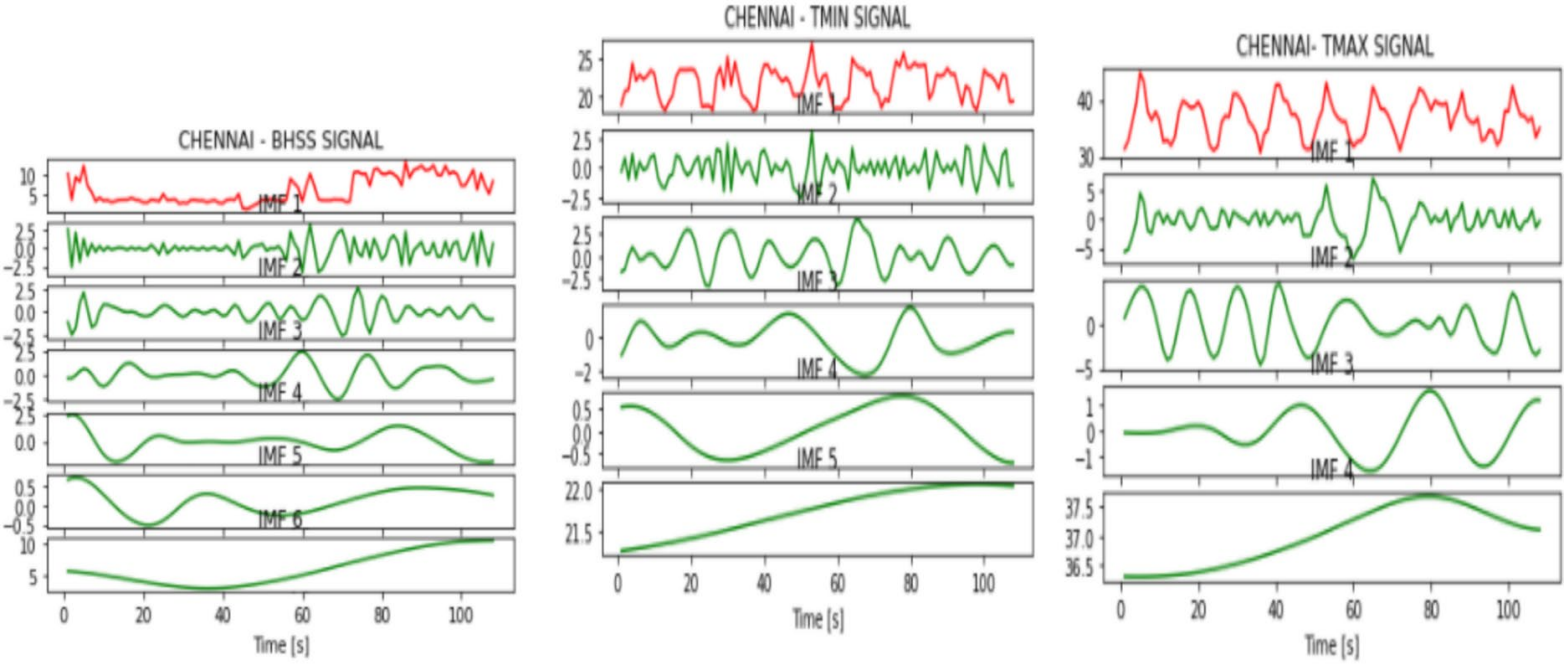
IMFs generated for Chennai using EEMD

**Fig. 11:**
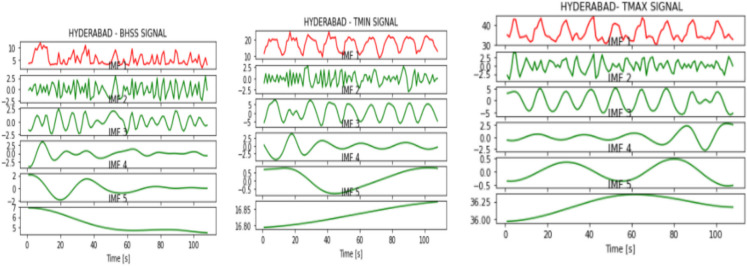
IMFs generated for Hyderabad using EEMD

**Fig. 12:**
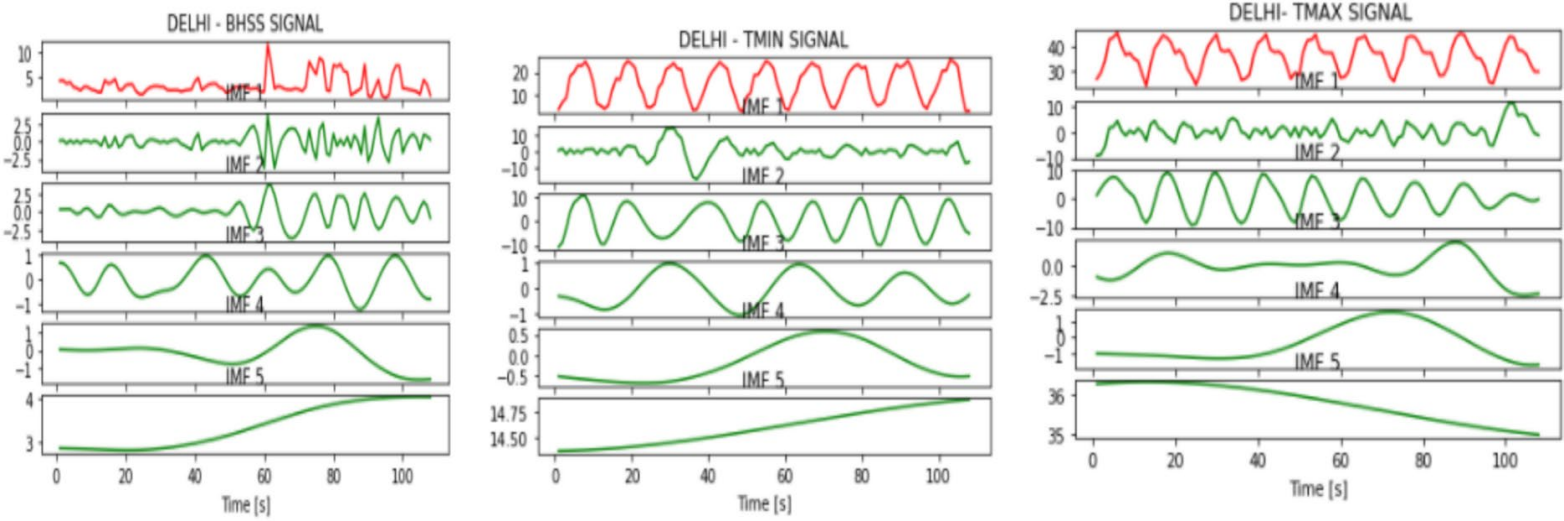
IMFs generated for Delhi using EEMD

**Fig. 13:**
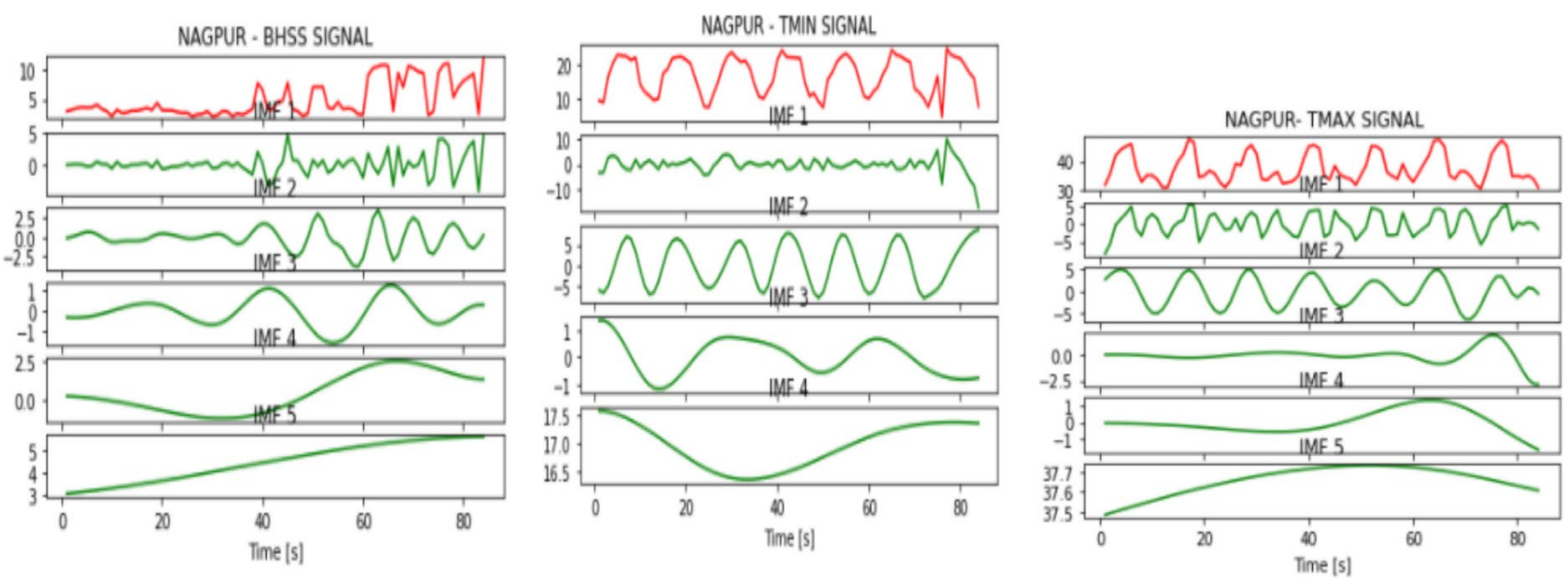
IMFs generated for Nagpur using EEMD

**Fig. 14:**
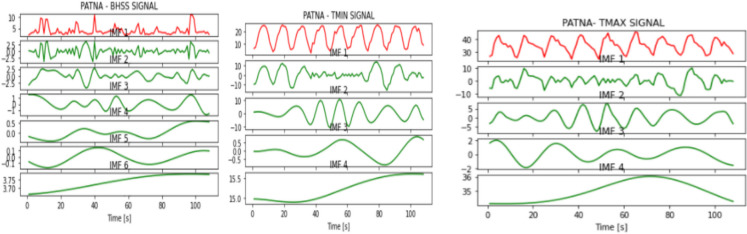
IMFs generated for Patna using EEMD

**Fig. 15:**
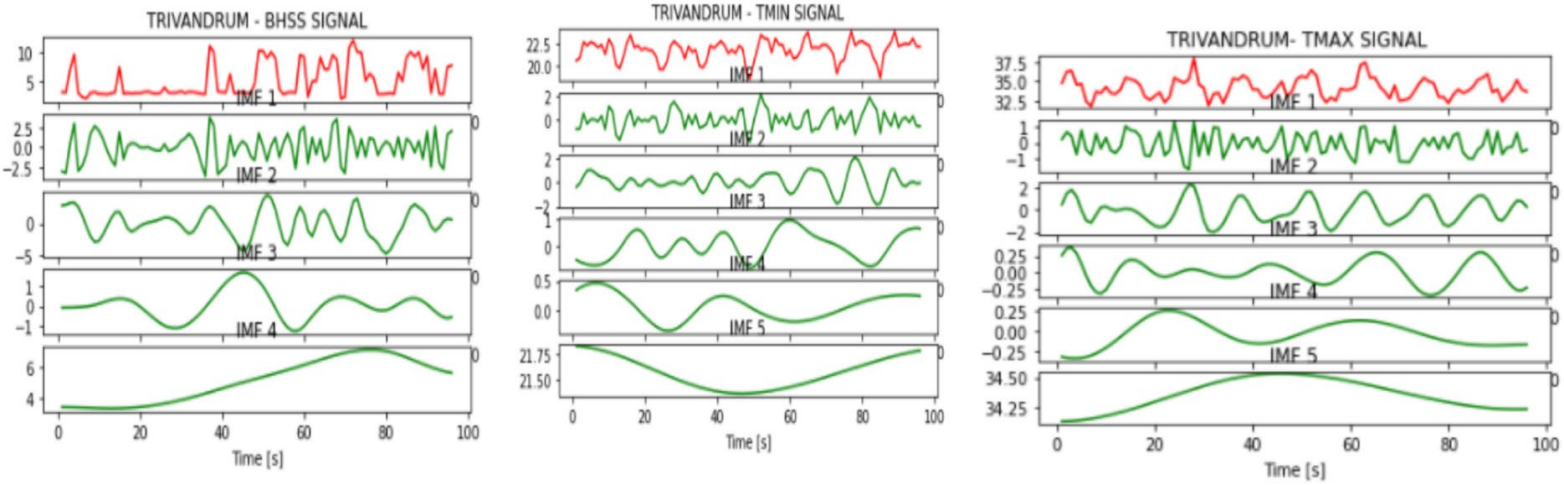
IMFs generated for Trivandrum using EEMD

Figures 9, 10, 11, 12, 13, 14, and 15 represent the IMFs generated for the seven states we have considered in this study by the EEMD technique.

### ML techniques

The role of predicting solar radiation is known as a supervised regression problem in the ML based methods. This is applied for the prediction of the outcome of an occurrence based on the continuous value of the relationship between variables extracted from datasets. The projected capacities of various ML regression techniques in this problem of solar radiation is analyzed and compared. The corresponding sections elaborate on the functional description of the four ML algorithms utilized in this work.

#### Ridge regression

The very first process in ridge regression is to normalize the variables (both dependent and independent) by dividing by their standard deviations and removing their mean values. All ridge regression computations are dependent on standardized variables in terms of standardization. The resulting regression coefficients are rescaled to their original when they are presented. It is used to measure the magnitude of coefficients in order to calculate data over fitting. The correlation coefficients demonstrate which independent variables that are highly associated with the dependent variable and with each other; if the measure of fit of the model is a small value, it means that the model is well matched to the dataset. A control term forces the learning algorithm to adjust the data and keep the weights as low as possible. The regulated terms have an “alpha” parameter that controls the regularization of the model, i.e., helps in reducing ranking variants. It is represented using Eq. (6).

6 $$A=Bc+E$$

In above equation, *A* represents the dependent variable(s), *B* denotes the independent variables, while *C* is the regression-based coefficients to be evaluated, and *E* depicts the error term. It allows a user to choose a range of regressor values that a user would not be able to use if you used least—square is used. Positively correlated variables can be combined, and ridge regression can be used to reduce multi-collinearity. In regression analysis, multi-collinearity refers to a strong connection between the explanatory variables. This is frequently the case when a high number of explanatory variables are used in the analysis by Shariff and Duzan (2018). If the multi-collinearity is high, even when the regressors are practically completely correlated, one or more regressors are effectively eliminated while utilizing the ridge method.

#### Multilayer perceptron

Multilayer perceptron (MLP) is a fundamental technique in machine learning (Kalogirou, 2001). It is structured as feedforward neural network composed of a series of inputs, hidden layers, and an output layer. Guermoui et al. (2020) analyzed this model based on time series data in which the model identifies the suitable input features. Their findings indicate that the MLP model can perform statistically well than the other models. Rabehi et al. (2018) analyzed the work of MLP in conjuction with boosted decision trees and linear regression models to select appropriate inputs within each regression model. Although there are several radiation prediction models, the current research indicates MLP regressor predicts multiple target variables and produces best results for continuous values.

#### Support vector regression

SVM was introduced in the year 1995 to complete classification problem. This is extended to the area of regression and estimation problem, therefore, is referred to as SVR. One of the important advantages of SVR is that the computational intricacy of a problem has no bearing on measure of input space. In addition, it has superior generalization capabilities with high predictive accuracy. In recent decades, solar radiation has been accurately predicted under various climatic conditions, for example, in humid and arid regions of China, by Almaghrabi et al. (2022), Singh et al. (2012), and Thombare et al. (2022). SVR creates an optimal hyper plane by projecting data for training into a 1-D feature set that also depicts the non-linear connection of inputs and outputs. SVR function is as mentioned in Eq. (7). The forecast results are denoted by ***f(y)***, ***F*** is the l-dimensional weight factor, ***a*** is an adjusting element, while $\boldsymbol{\varphi }$ ***(y)*** depicts the map function of projecting into the high l-D feature set. SVR has been proven to be an effective tool in real-value function estimation.

7 $${\varvec{f}}\left({\varvec{y}}\right)={\varvec{F}}\boldsymbol{\varphi }\left({\varvec{y}}\right)+{\varvec{a}}$$

#### Random forest regression

The RFR model is an additive based model that creates forecasts through combining the results with several different models. As a forecast value, it uses the mean or median of all outputs. This type of models can be written more explicitly as Eq. (8).

8 $$F\left(x\right)={\text{g}}_{0}\left(x\right)+{\text{g}}_{1}\left(x\right){\text{g}}_{2}\left(x\right)+... {\text{g}}_{n}\left(x\right)$$

where ***F(x)*** is the addition of simpler core models “**g**_**i**_” where “i” iterates from 1 to *n*, *n* being the highest count of models. In this method, all baselines are created independently with different subsample of the data. Although, a large number of trees can produce high computational costs and takes up a lot of memory, and even slower predictions, which can lead to challenges, but they are parallelizable, which means that we can do this around the process of dividing several machines which will run. This leads to faster computation time. Because of lower parameter settings and faster applications, this also performs better than other ML methods (Belgiu & Drăguţ, 2016). So, in our research, we considered RFR which also helps overcome missing values and maintains the accuracy as the solar radiation for a long-term data is estimated.

## Results

In the proposed model, EEMD is often preferred for its robust decomposition capabilities, effectively addressing the “mode mixing” issue prevalent in EMD. It strikes a favorable balance between computational complexity and accuracy, particularly in noisy signal scenarios. A comparative analysis is done between empirical mode decomposition (EMD) and ensemble empirical mode decomposition (EEMD) to which the input data features such as *T*max, *T*min, and bright sunshine hours are taken from top metropolitan cities in India. The output value from both EMD and EEMD will undergo four ML models for calculating statistical errors such as RMSE, MAE, and *R* measured individually for every metropolitan stations. Among the four ML models, it is observed that when evaluated individually for each metric, the RFR and RIDGE models had given a good accuracy as well but when computed in terms of the performance metrics taken as a whole, the MLP models had a best accuracy. In order to implement the MLP, based on trial and error process, the hidden layers count was determined. The count for hidden layer was not fixed for any of the locations. For instance, for Hyderabad, six counts were used; whereas for Patna and Trivandrum, five counts were used. Here, activation functions used were tangent (tanh) and logistic and rectified linear unit (relu) respectively. For optimization limited-memory Broyden–Fletcher–Goldfarb–Shanno (lbfgs), stochastic GD (SGD), and Adam methods were used. As a result, the MLP models (on average RMSE of EMD: 0.306 and EEMD: 0.332, MAE of EMD: 0.27 and EEMD: 0.26, and *R* of EMD: 0.9919 and EEMD: 0.9941) exhibit the best prediction accuracy among the eight ML models at each station. Among the MLP-EMD and MLP-EEMD models, in terms of the metrics MAE and *R*, the MLP-EEMD has acquired the best results and when evaluated in terms of RMSE the MLP-EMD has yielded the best results.

**Fig. 16:**
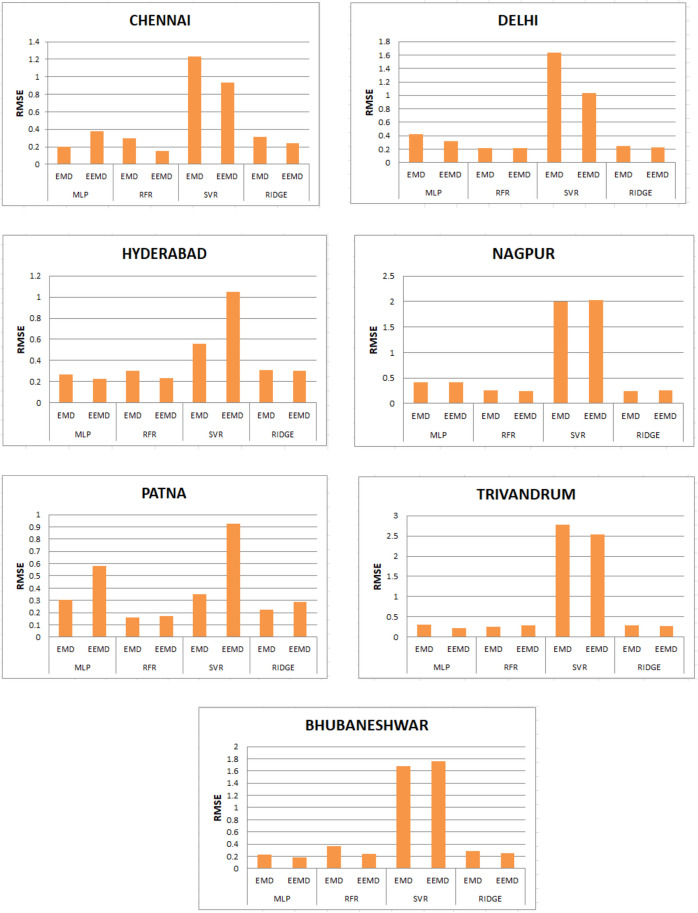
Histograms for RMSE values obtained for the seven states using EMD and EEMD

**Fig. 17:**
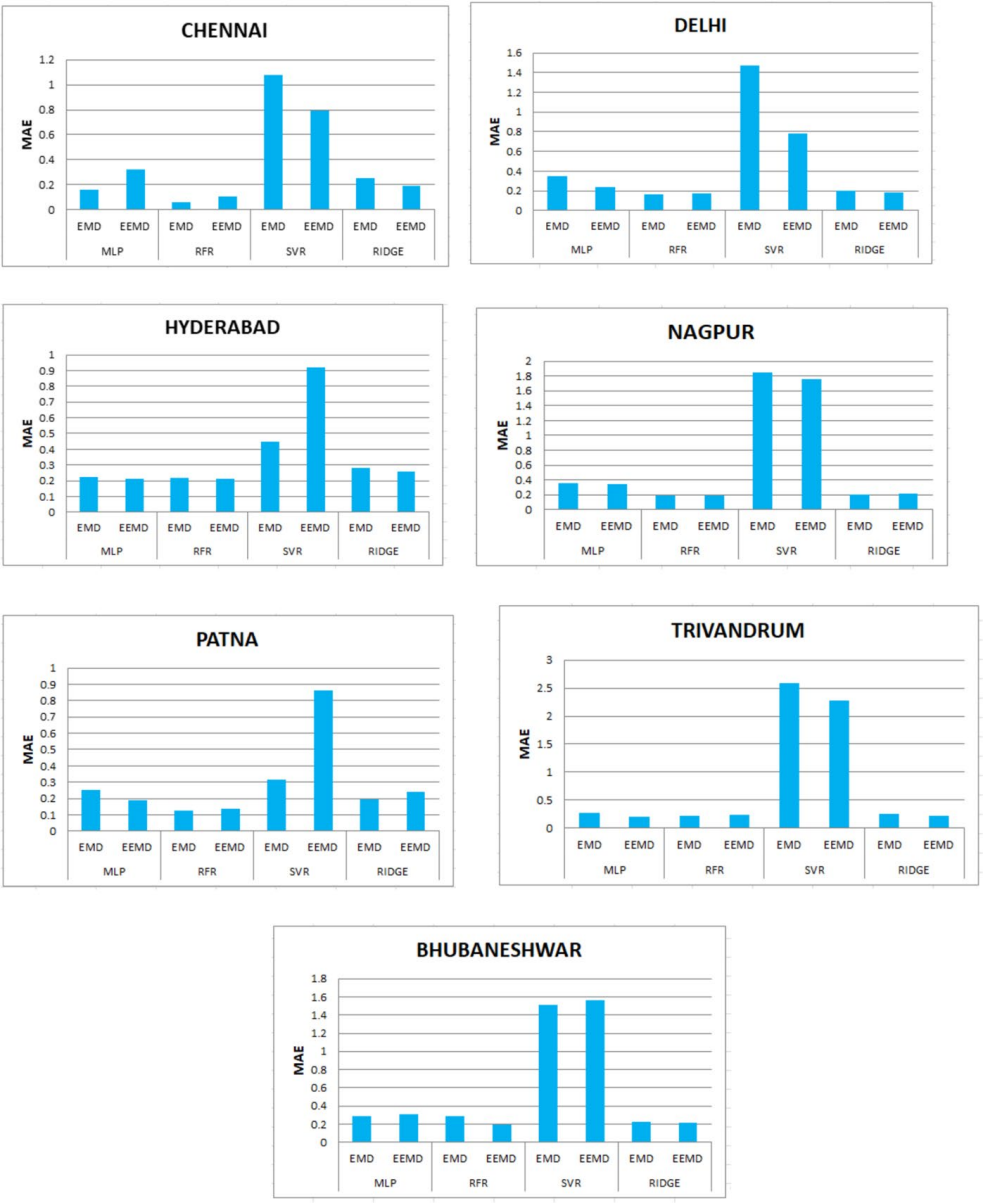
Histograms for MAE values obtained for the seven states using EMD and EEMD

The RFR-EMD model ranks as the next optimum model which is succeeded by RIDGE-EEMD. However, the SVR-EEMD model gives the lowest accuracy. The eight models trained and evaluated for RMSE in Table 2, and the diagrammatic representation is shown in Fig. 16. Similarily MAE evaluation and diagrammatic representation is shown in Fig. 17. As in the preceding situation, the MLP-EEMD model (on average RMSE of 0.306) provides the best prediction accuracy with regard to the eight models. It has its ranking in the order 0.204 for Chennai, 0.23 for Bhubaneswar, 0.266 for Hyderabad, 0.3 for Trivandrum, 0.3028 for Patna, 0.42 for Nagpur, and 0.421 for Delhi. But as stated previously, RFR and RIDGE given better results as well with the RFR-EEMD acquiring an average RMSE: 0.221 and RIDGE: 0.263. The SVR model has given the lowest accurate values when compared with the other three models. Same as previous metric evaluation, the MLP has yielded best accuracy. But as previously stated, the MLP-EEMD has outperformed the MLP-EMD with an MAE: 0.26. It has its ranking in the order: Patna (0.19), Trivandrum (0.202), Hyderabad (0.212), Delhi (0.241), Bhubaneshwar (0.31), Chennai (0.324), and Nagpur (0.3439). RFR and RIDGE have given better results as well. But for this metric RFR-EMD has given the better results with an average RMSE: 0.1811 and RIDGE-EEMD: 0.218. The SVR-EMD model has given the lowest accurate values when compared with the other three models.

**Table 2:** RMSE values obtained for the seven states using EMD and EEMD

City	MLP		RFR		SVR		RIDGE	
	EMD	EEMD	EMD	EEMD	EMD	EEMD	EMD	EEMD
Chennai	0.204	0.381	0.3	0.15	1.23	0.93	0.317	0.24
Delhi	0.421	0.321	0.211	0.22	1.636	1.03	0.245	0.228
Hyderabad	0.266	0.227	0.301	0.234	0.56	1.05	0.31	0.3
Nagpur	0.42	0.416	0.252	0.239	2	2.03	0.247	0.262
Patna	0.303	0.58	0.159	0.17	0.349	0.924	0.225	0.287
Trivandrum	0.3	0.22	0.261	0.289	2.78	2.53	0.288	0.267
Bhubaneshwar	0.23	0.18	0.37	0.243	1.68	1.76	0.285	0.257

Table 3 and Fig. 17 depicts the performance metrics of various machine learning algorithms integrated with EMD and EEMD for solar radiation forecasting are summarized across several cities. In Chennai, the MLP model using EMD achieved the lowest RMSE of 0.158, while the EEMD integration resulted in a higher RMSE of 0.324. For random forest regression (RFR), the EMD model produced an RMSE of 0.064, significantly lower than the EEMD’s 0.1055. In Delhi, the EMD-based MLP showed an RMSE of 0.346, compared to 0.241 with EEMD, whereas RFR delivered 0.163 for EMD and 0.1729 for EEMD. Hyderabad’s models displayed closely matched results, with MLP achieving 0.225 for EMD and 0.212 for EEMD. In Nagpur, RFR performed similarly well, yielding 0.1962 for EMD and 0.1957 for EEMD. Patna exhibited the best performance overall with RFR under EMD achieving the lowest RMSE of 0.126, while EEMD reached 0.14. Trivandrum and Bhubaneswar showed varied results, with Trivandrum’s MLP yielding an RMSE of 0.27 (EMD) and 0.202 (EEMD), and Bhubaneswar’s RFR producing 0.285 for EMD and 0.2 for EEMD. Overall, these results indicate significant variations in model performance depending on both the machine learning approach and the data processing technique used.

**Table 3:** MAE values obtained for the seven states using EMD and EEMD

City	MLP		RFR		SVR		RIDGE	
	EMD	EEMD	EMD	EEMD	EMD	EEMD	EMD	EEMD
Chennai	0.158	0.324	0.064	0.1055	1.08	0.792	0.25	0.19
Delhi	0.346	0.241	0.163	0.1729	1.47	0.779	0.2002	0.1816
Hyderabad	0.225	0.212	0.218	0.213	0.448	0.921	0.28	0.26
Nagpur	0.359	0.3439	0.1962	0.1957	1.847	1.76	0.2052	0.21723
Patna	0.2551	0.19	0.126	0.14	0.3158	0.862	0.1927	0.2426
Trivandrum	0.27	0.202	0.216	0.245	2.59	2.28	0.255	0.227
Bhubaneshwar	0.284	0.31	0.285	0.2	1.51	1.56	0.2231	0.211

**Fig. 18:**
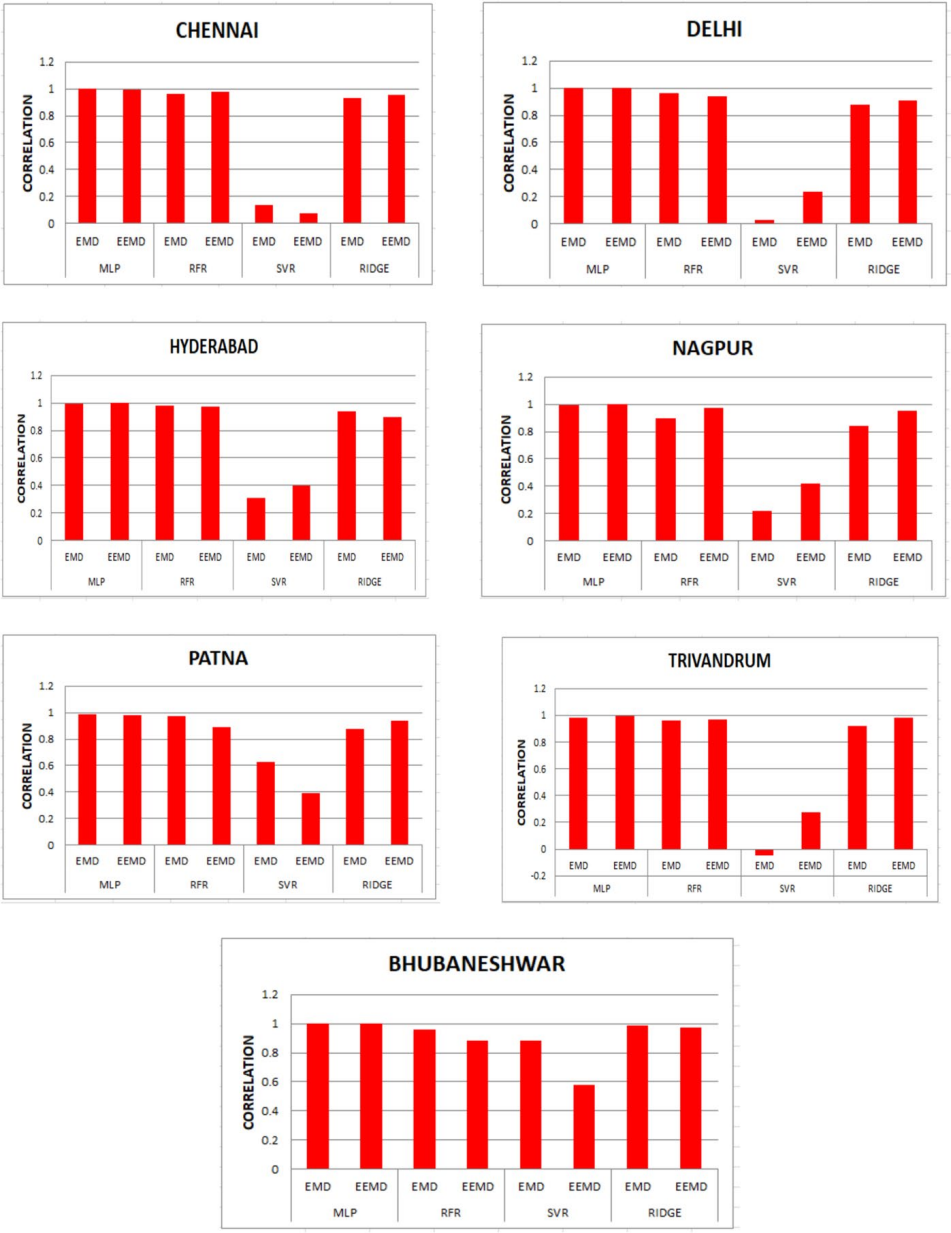
Histograms for correlation coefficient (*R*) values obtained for the seven states using EMD and EEMD

**Fig. 19:**
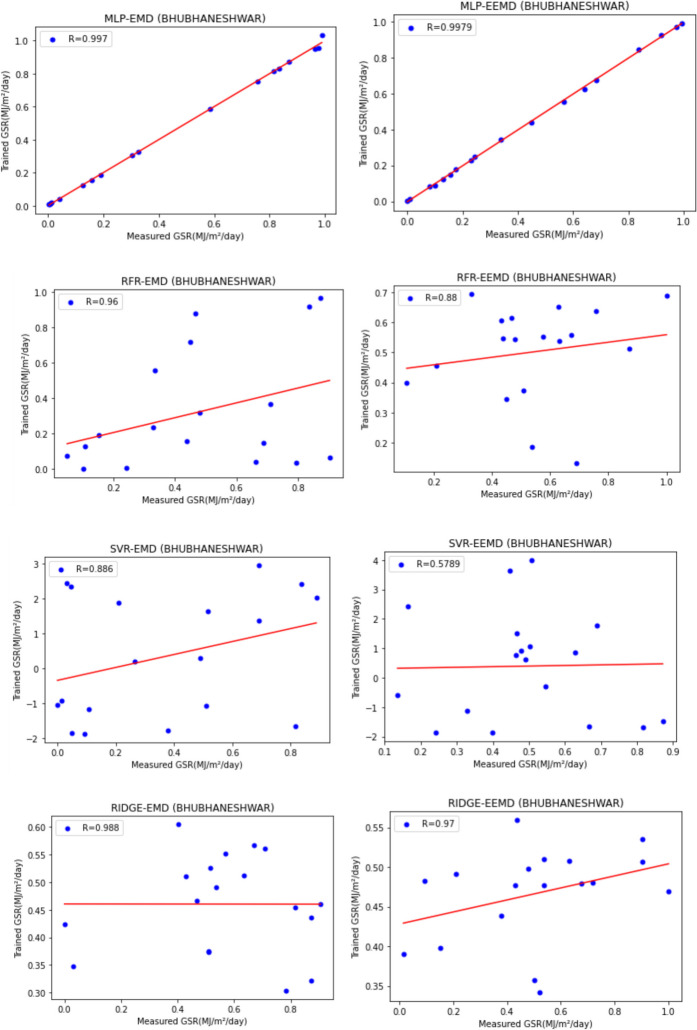
Scatter plots for Bhubaneshwar based on the eight models

**Fig. 20:**
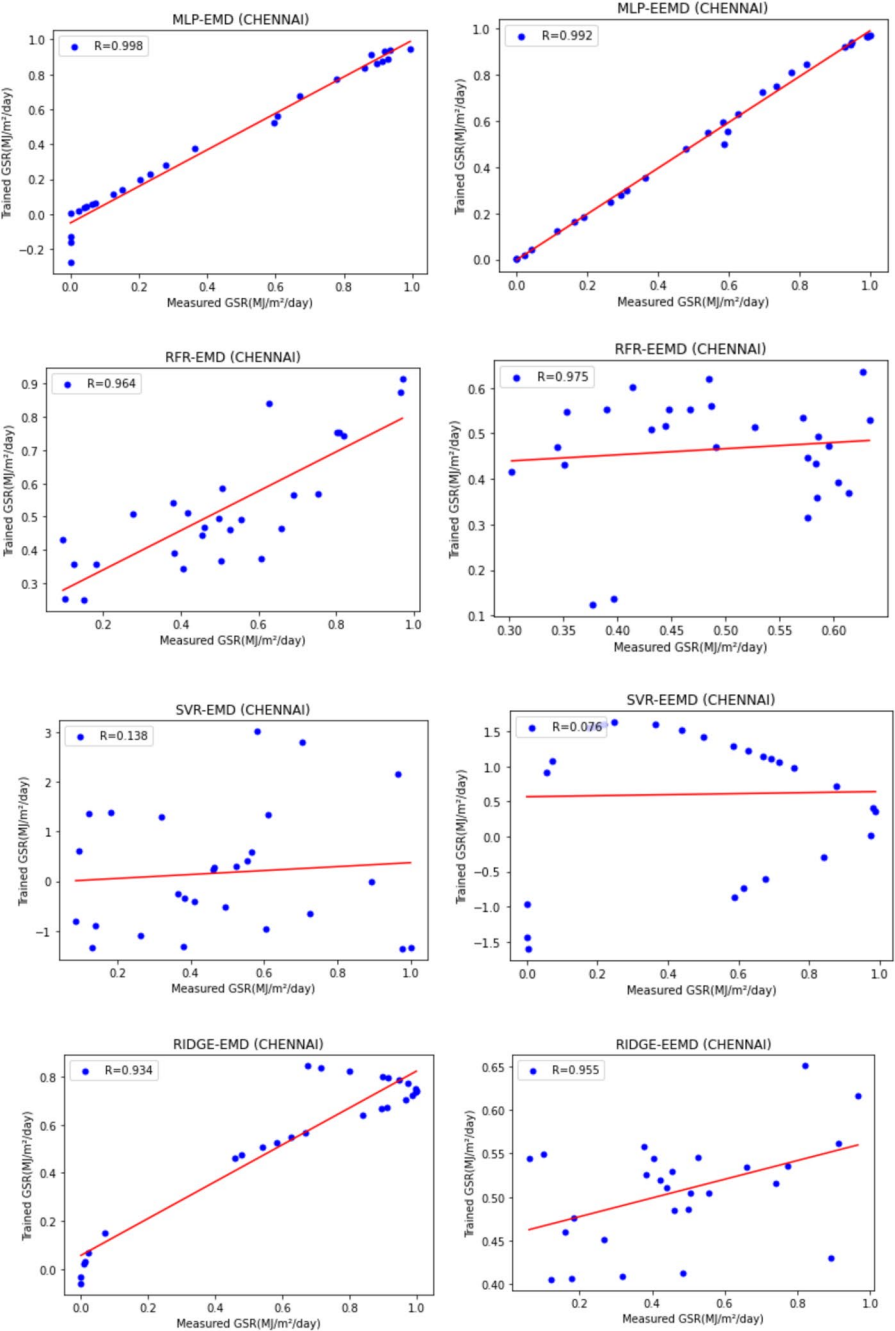
Scatter plots for Chennai based on the eight models

**Fig. 21:**
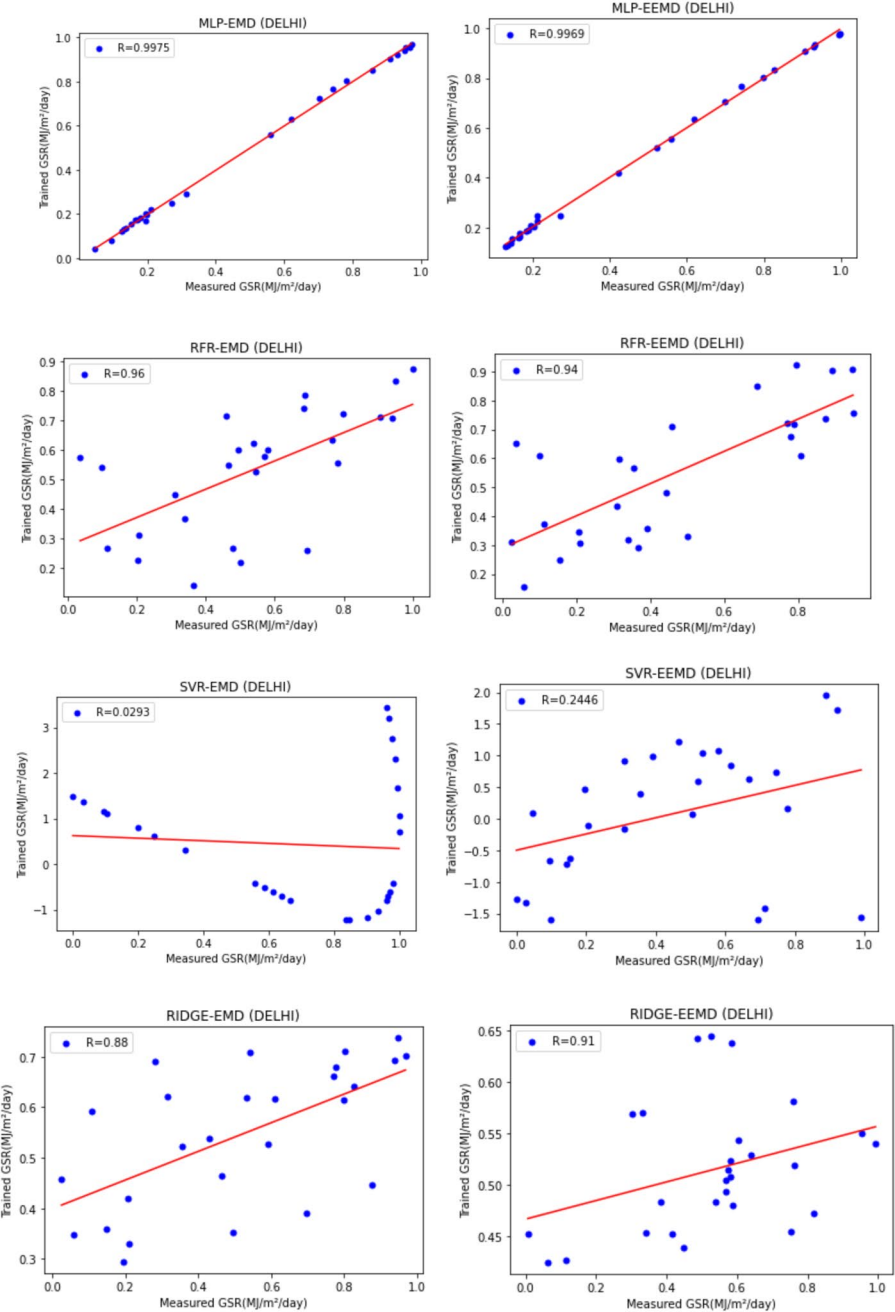
Scatter plots for Delhi based on the eight models

**Fig. 22:**
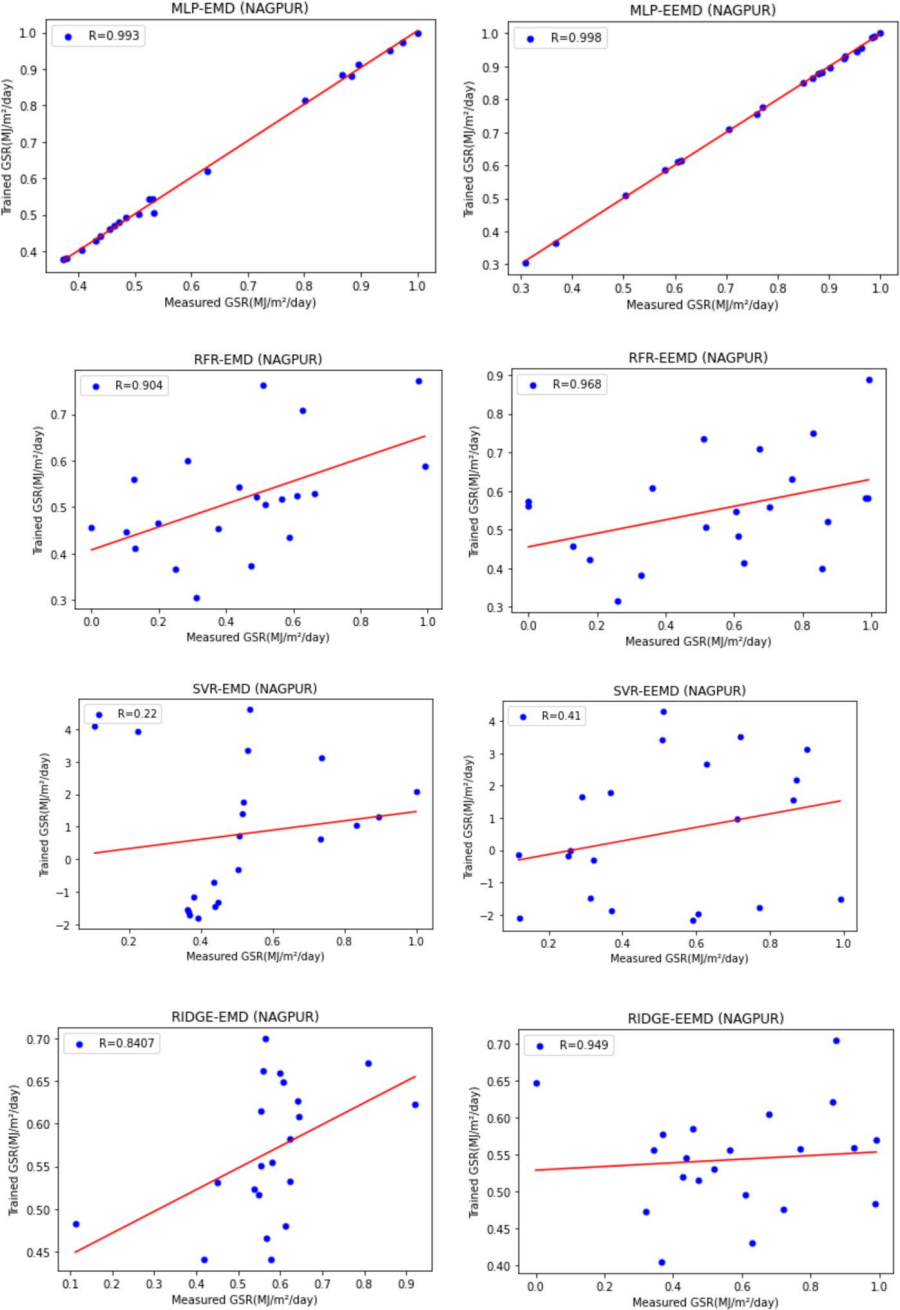
Scatter plots for Nagpur based on the eight models

**Fig. 23:**
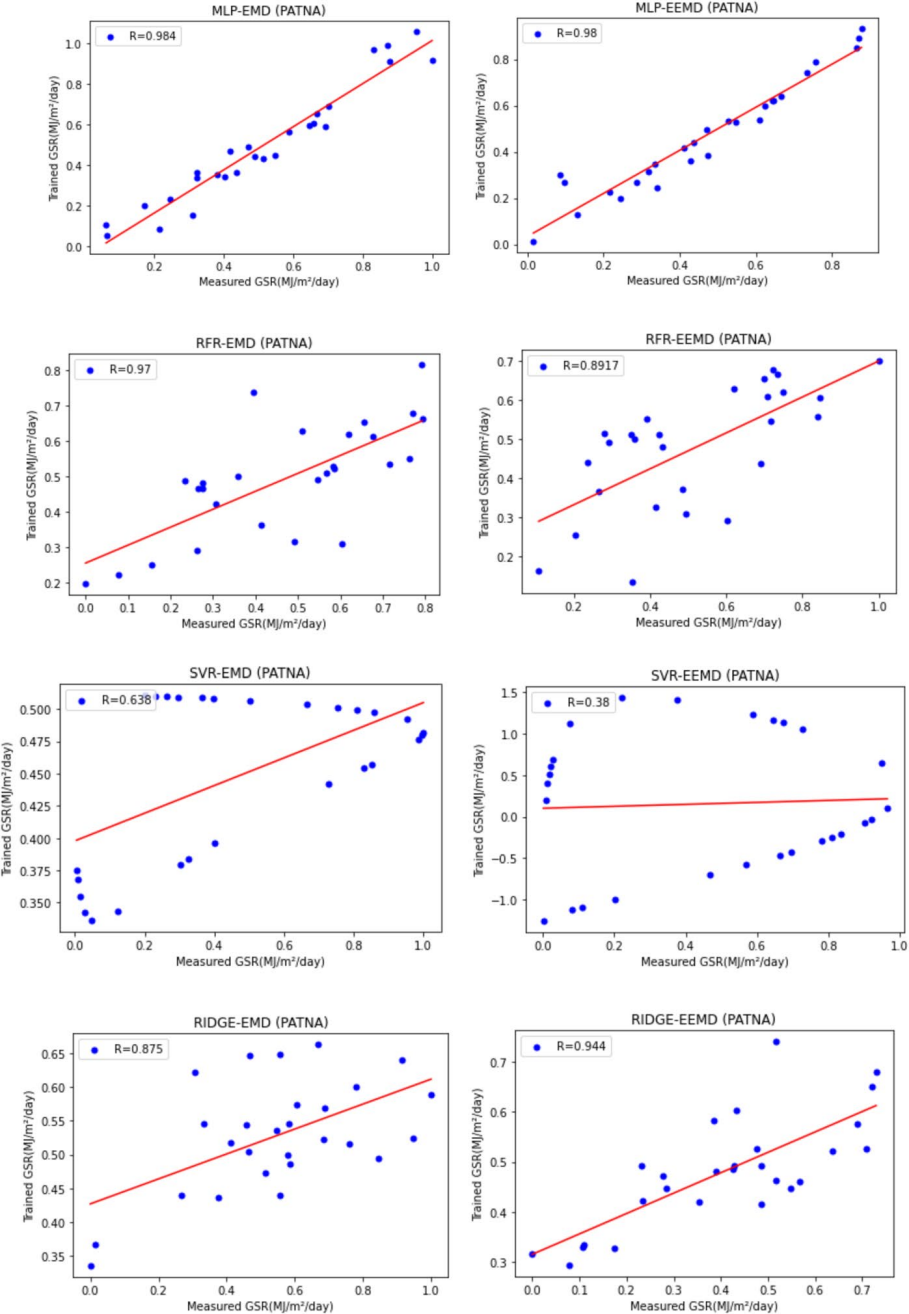
Scatter plots for Patna based on the eight models

**Fig. 24:**
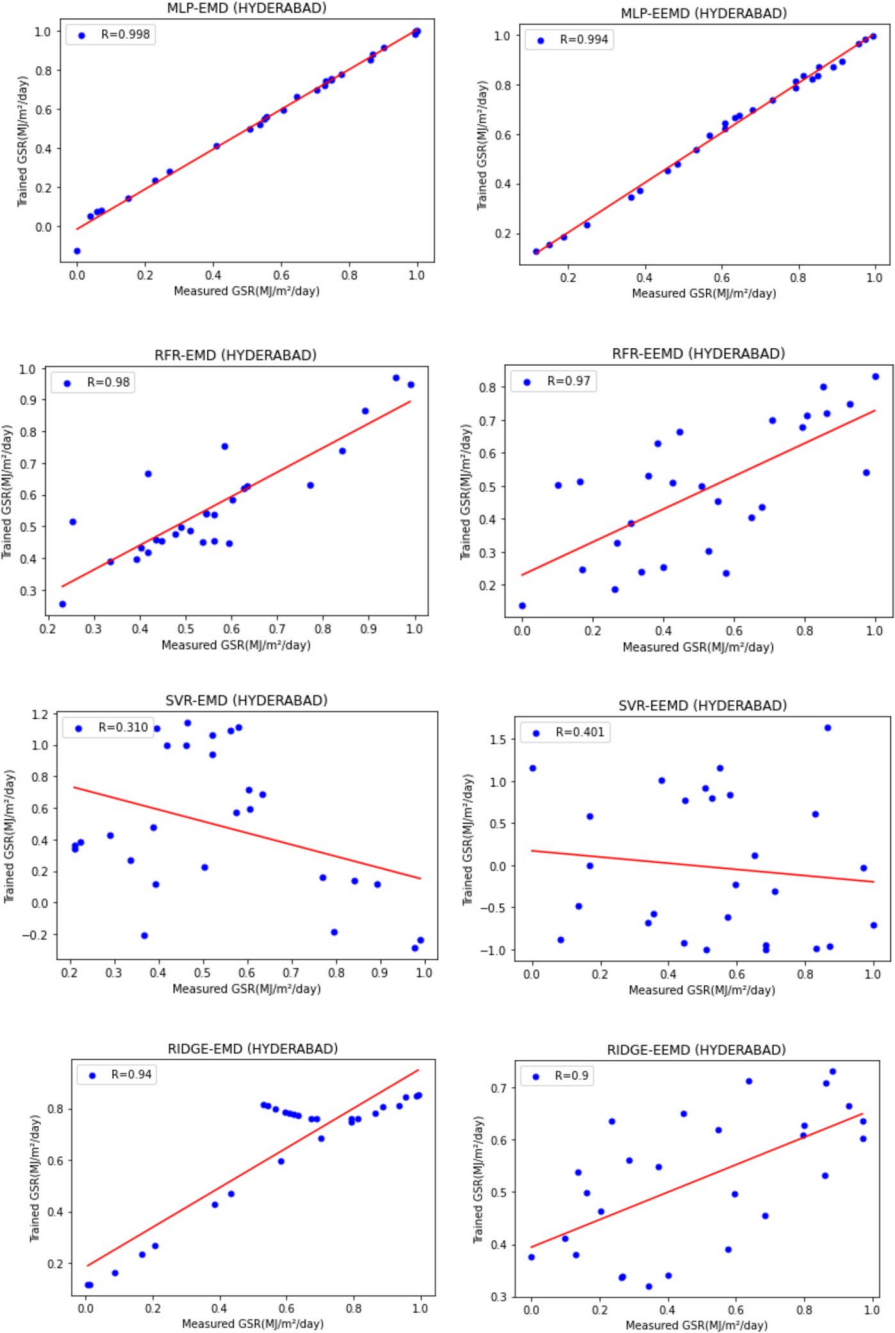
Scatter plots for Hyderabad based on the eight models

**Fig. 25:**
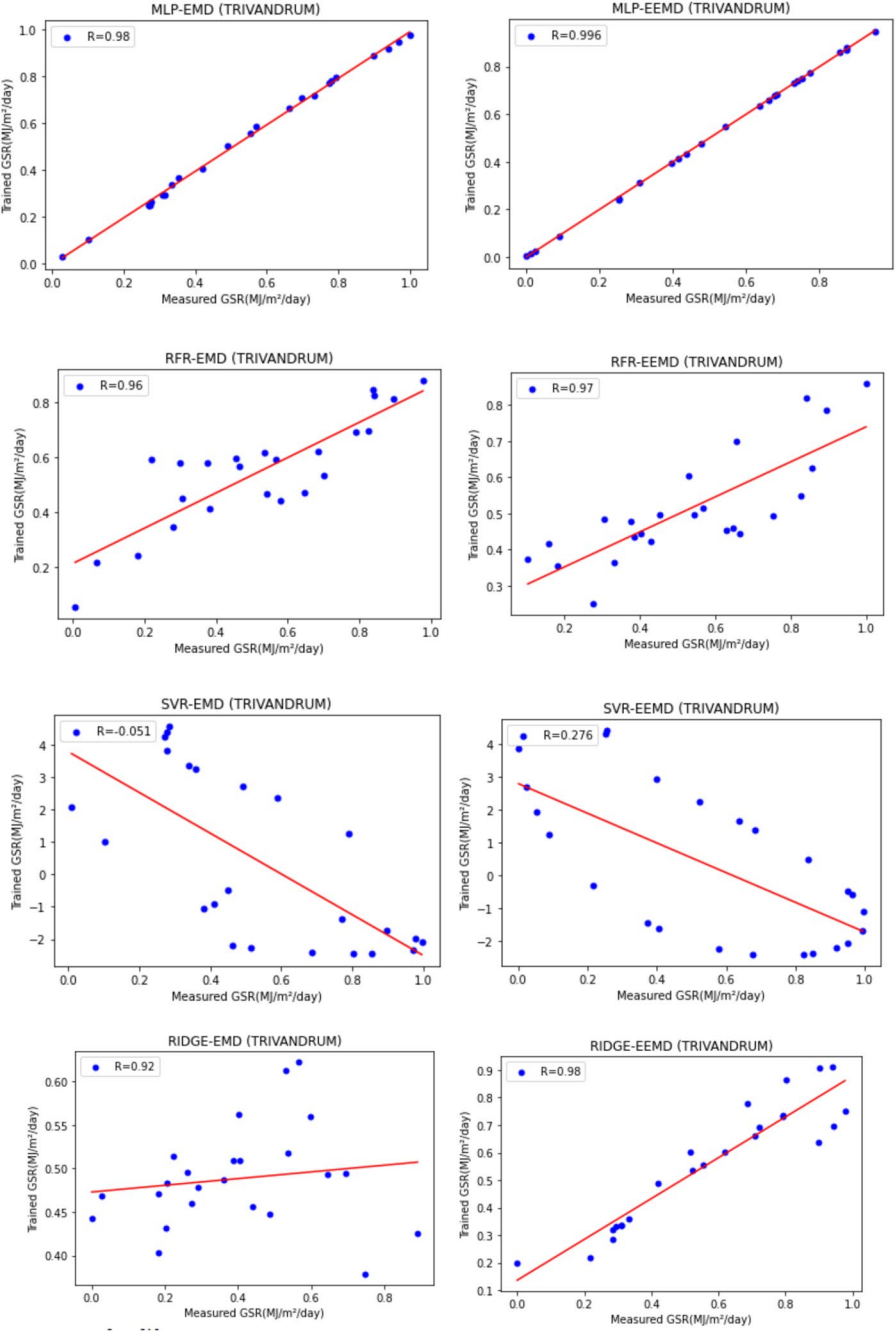
Scatter plots for Trivandrum based on the eight models

**Fig. 26:**
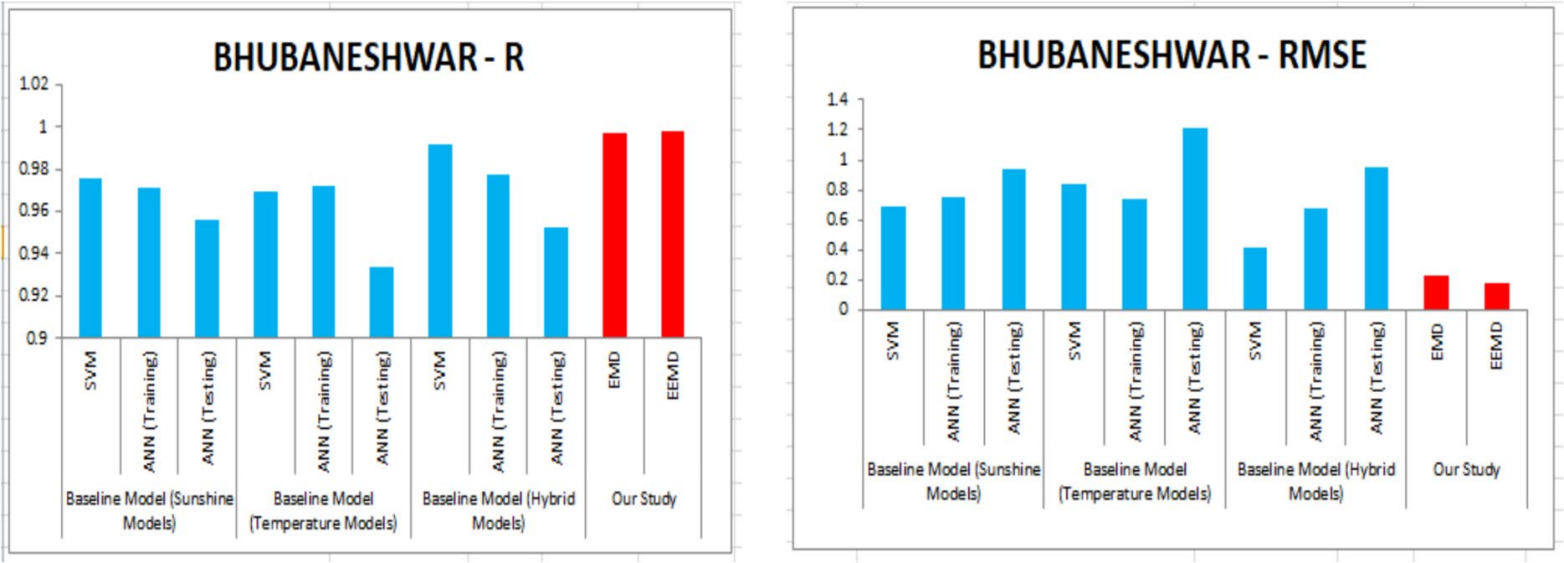
*R* and RMSE comparison of our study vs. the baseline models for Bhubaneshwar

**Fig. 27:**
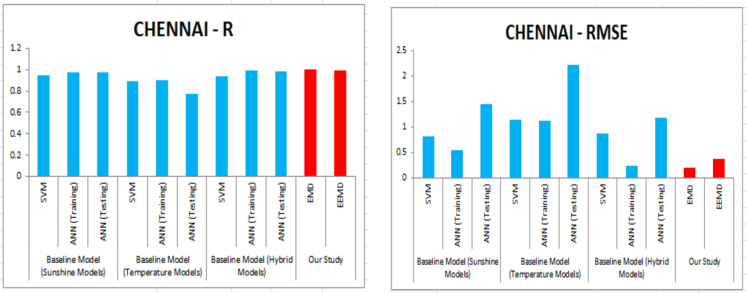
*R* and RMSE comparison of our study vs. the baseline models for Chennai

**Fig. 28:**
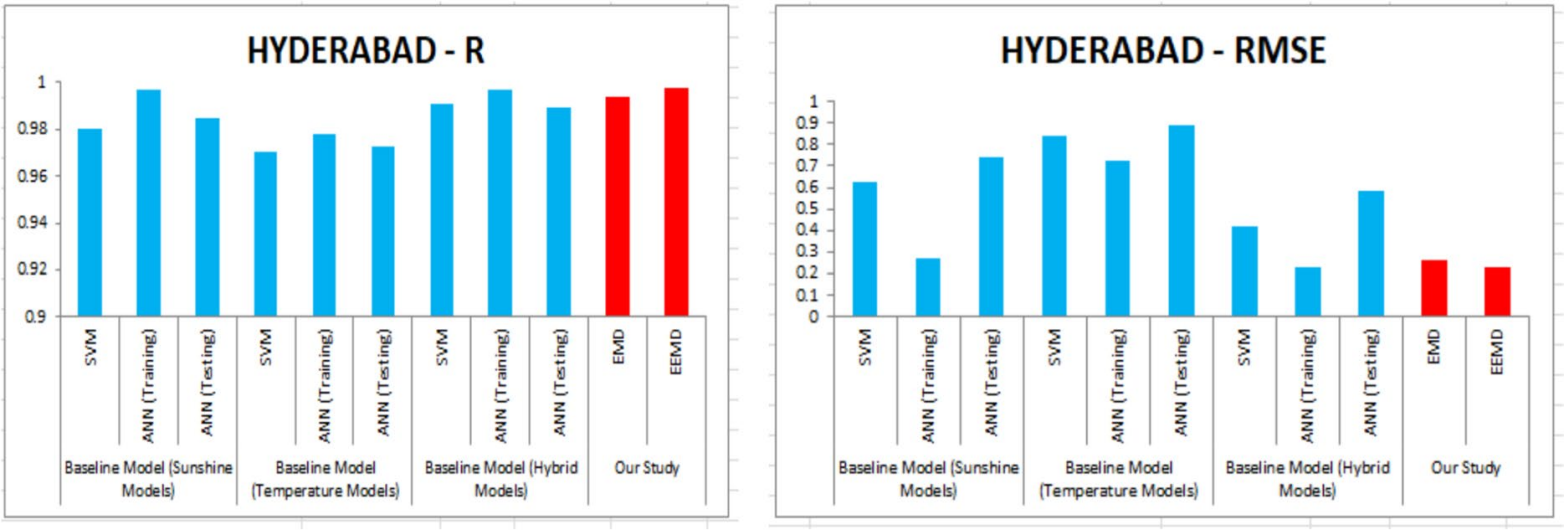
*R* and RMSE comparison of our study vs. the baseline models for Hyderabad

**Fig. 29:**
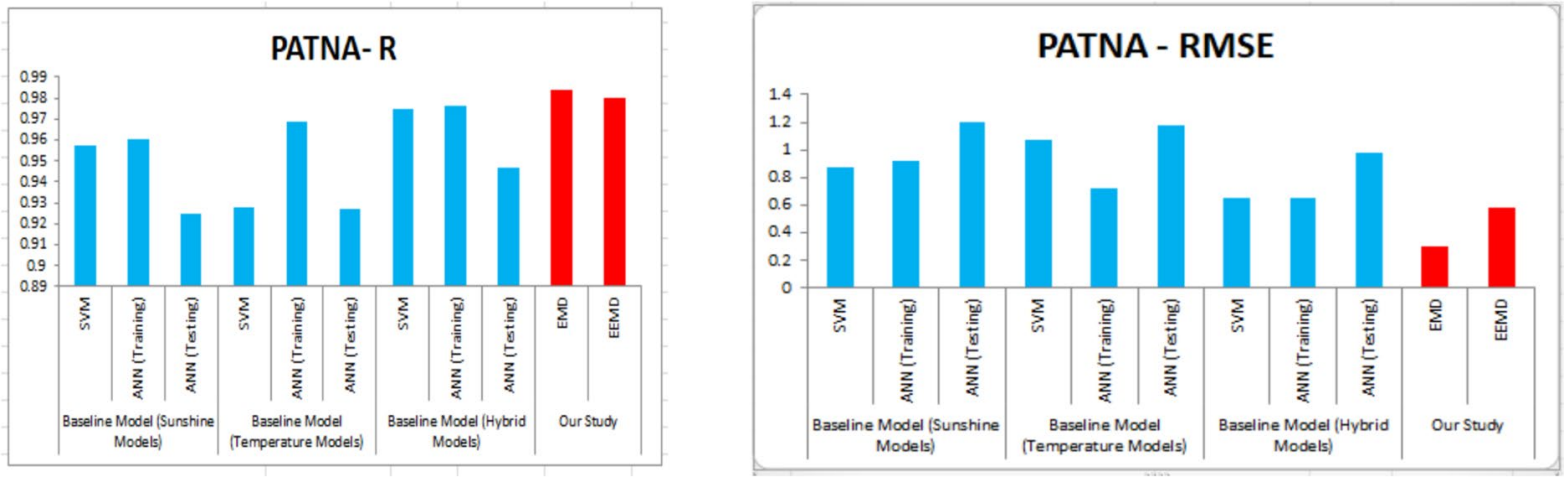
*R* and RMSE comparison of our study vs. the baseline models for Patna

For our work, we have mainly considered the paper (Meenal & Selvakumar, 2018) as our baseline model. Meenal and Selvakumar 2018 have applied the SVM and ANN for the GSR forecast. The eight models trained and evaluated for *R* in Table 4, and the diagrammatic representation is shown in Figs. 18, 19, 20, 21, 22, 23, 24 and 25. Tables 5, 6, 7, and 8 depict the performance of our study with the baseline model for Bhubaneshwar, Chennai, Hyderabad, and Patna. Figures 26, 27, 28, and 29 depict the *R* and RMSE values comparison obtained using our study and the baseline model.

**Table 4:** Correlation coefficient (*R*) values obtained for the seven states using EMD and EEMD

City	MLP		RFR		SVR		RIDGE	
	EMD	EEMD	EMD	EEMD	EMD	EEMD	EMD	EEMD
Chennai	0.998	0.992	0.964	0.975	0.14	0.076	0.93	0.955
Delhi	0.9975	0.9969	0.96	0.94	0.029	0.24	0.88	0.91
Hyderabad	0.994	0.998	0.98	0.97	0.31	0.4	0.94	0.9
Nagpur	0.993	0.998	0.9	0.97	0.22	0.42	0.84	0.95
Patna	0.984	0.98	0.97	0.89	0.63	0.39	0.875	0.94
Trivandrum	0.98	0.996	0.96	0.97	−0.051	0.276	0.92	0.98
Bhubaneshwar	0.997	0.9979	0.96	0.88	0.886	0.58	0.988	0.97

**Table 5:** Comparison of our study vs. the baseline model for *R* and RMSE values for Bhubaneshwar

		*R*	RMSE
Baseline model (sunshine models)	SVM	0.9756	0.6886
	ANN (training)	0.9711	0.7466
	ANN (testing)	0.9559	0.9377
Baseline model (temperature models)	SVM	0.9697	0.8354
	ANN (training)	0.9723	0.7408
	ANN (testing)	0.9334	1.2112
Baseline model (hybrid models)	SVM	0.9919	0.4223
	ANN (training)	0.977	0.68
	ANN (testing)	0.952	0.9553
Our study	EMD	0.997	0.23
	EEMD	0.9979	0.18

**Table 6:** Comparison of our study vs. the baseline model *R* and RMSE values for Chennai

		*R*	RMSE
Baseline model (sunshine models)	SVM	0.9465	0.8083
	ANN (training)	0.9784	0.5437
	ANN (testing)	0.9739	1.4468
Baseline model (temperature models)	SVM	0.8881	1.1434
	ANN (training)	0.9043	1.1199
	ANN (testing)	0.7727	2.2197
Baseline model (hybrid models)	SVM	0.9365	0.8673
	ANN (training)	0.9963	0.2472
	ANN (testing)	0.9835	1.1848
Our study	EMD	0.998	0.204
	EEMD	0.992	0.381

**Table 7:** Comparison of our study vs. the baseline model *R* and RMSE values for Hyderabad

		*R*	RMSE
Baseline model (sunshine models)	SVM	0.9805	0.6278
	ANN (training)	0.9968	0.2722
	ANN (testing)	0.9847	0.7451
Baseline model (temperature models)	SVM	0.9704	0.8384
	ANN (training)	0.9782	0.7212
	ANN (testing)	0.9725	0.8929
Baseline model (hybrid models)	SVM	0.9911	0.4205
	ANN (training)	0.9968	0.229
	ANN (testing)	0.9894	0.5814
Our study	EMD	0.994	0.266
	EEMD	0.998	0.227

**Table 8:** Comparison of our study vs. the baseline model *R* and RMSE values for Patna

Baseline model (sunshine models)	SVM	0.9573	0.8735
	ANN (training)	0.9601	0.9214
	ANN (testing)	0.9244	1.2003
Baseline model (temperature models)	SVM	0.9277	1.0769
	ANN (training)	0.969	0.7192
	ANN (testing)	0.927	1.1807
Baseline model (hybrid models)	SVM	0.9746	0.6515
	ANN (training)	0.9765	0.6508
	ANN (testing)	0.9465	0.9828
Our study	EMD	0.984	0.3028
	EEMD	0.98	0.58

Table 4 depicts the correlation coefficient values generated by the eight models. The maximum correlation coefficient was yielded by the MLP-EEMD model with an average value of 0.9941 followed by MLP-EMD (0.9919), RFR-EMD (0.956), RIDGE-EEMD (0.943), RFR-EEMD (0.942), RIDGE-EMD (0.91), SVR-EEMD (0.34), and SVR-EMD (0.31) respectively in the order. The MLP-EEMD model has correlation co-efficient ranked among the selected cities as follows: Hyderabad and Nagpur (0.998), Bhubaneswar (0.9979), Delhi (0.9969) Trivandrum (0.996), Chennai (0.992), and Patna (0.98). Figure 18 shows the visual representation of *R* for all the stations. Hence from the comparisons, it is found that MLP models have the best and SVR models have the least forecasting capability with least error using the selected three features—${T}_{\text{min}}$, ${T}_{\text{max}}$, and BHSS. This could additionally be visually in terms of scatter plots that represent the closeness of values predicted by MLP vs. other algorithms.

Figures 19, 20, 21, 22, 23, 24, and 25 represent the variation between the values obtained for *R* by the machine learning models applied in our study for the seven states. It is observed that MLP has the best performance.

For our work, we mainly considered Perveen et al. (2018) as our baseline model. To estimate the global solar radiation (GSR), few models have built for representing its empirical relationship. For our work, we have used sunshine model, temperature model and hybrid model as a baseline model. Table 5 depicts the training error of RMSE and *R* values for Bhuvaneshwar. Comparing EMD and EEMD with ANN, it is observed that the model EEMD yield best result. Perveen et al. (2018) have applied the SVM and ANN for the GSR forecast. Similarly Tables 6, 7, and 8 depict the performance of our study with the baseline model for Chennai, Hyderabad, and Patna. Figures 26, 27, 28, and 29 depict the *R* and RMSE values comparison obtained using our study and the baseline model.

The solar radiation is forecasted for the seven states by applying the SVM technique by the authors of our baseline model and generating multiple SVM models. Based on the performance of the SVM models, they have been allocated a rank. When compared with the SVM model ranked as best in the baseline paper, our study has acquired better results using MLP. Table 9 illustrates the comparison of the results obtained in our study with the baseline model.

**Table 9:** Comparison table of baseline models vs. our study using *R* and RMSE

City			*R*	RMSE
Bhubaneshwar	Baseline model	SVM	0.9919	0.4223
	Our study	MLP-EMD	0.997	0.3
		MLP-EEMD	0.9979	0.22
Chennai	Baseline model	SVM	0.9465	0.8083
	Our study	MLP-EMD	0.998	0.204
		MLP-EEMD	0.992	0.381
Hyderabad	Baseline model	SVM	0.9911	0.4205
	Our study	MLP-EMD	0.998	0.266
		MLP-EEMD	0.994	0.227
Patna	Baseline model	SVM	0.9746	0.6515
	Our study	MLP-EMD	0.984	0.3028
		MLP-EEMD	0.98	0.58
Delhi	Our study	MLP-EMD	0.9975	0.421
		MLP-EEMD	0.9969	0.321
Nagpur	Our study	MLP-EMD	0.993	0.42
		MLP-EEMD	0.998	0.4156
Trivandrum	Our study	MLP-EMD	0.98	0.3
		MLP-EEMD	0.996	0.22

Perveen et al. (2018) have applied the ANN technique for the solar forecast of the seven states which is illustrated in Table 10. The authors have applied the ANN technique for the sunshine model, temperature model, and the hybrid models. Our study has indicated better results when applied with MLP for all the outcomes yielded by the baseline model.

**Table 10:** State wise comparison of RMSE values with all algorithms used in our study vs. baseline models (ANN)

City	MLP		RFR		SVR		RIDGE		ANN (baseline-sunshine model)		ANN (baseline-temperature model)		ANN (baseline-hybrid model)	
	EMD	EEMD	EMD	EEMD	EMD	EEMD	EMD	EEMD	Training	Testing	Training	Testing	Training	Testing
Chennai	0.204	0.381	0.3	0.1502	1.23	0.93	0.317	0.24	0.5437	1.4468	1.1199	2.2197	0.2472	1.1848
Hyderabad	0.266	0.227	0.301	0.234	0.56	1.05	0.31	0.3	0.2722	0.7451	0.7212	0.8929	0.229	0.5814
Patna	0.3028	0.58	0.159	0.17	0.3488	0.924	0.225	0.2868	0.9214	1.2003	0.7192	1.1807	0.6508	0.9828
Bhubaneshwar	0.23	0.18	0.37	0.243	1.68	1.76	0.285	0.257	0.7466	0.9377	0.7408	1.2112	0.68	0.9553

**Fig. 30:**
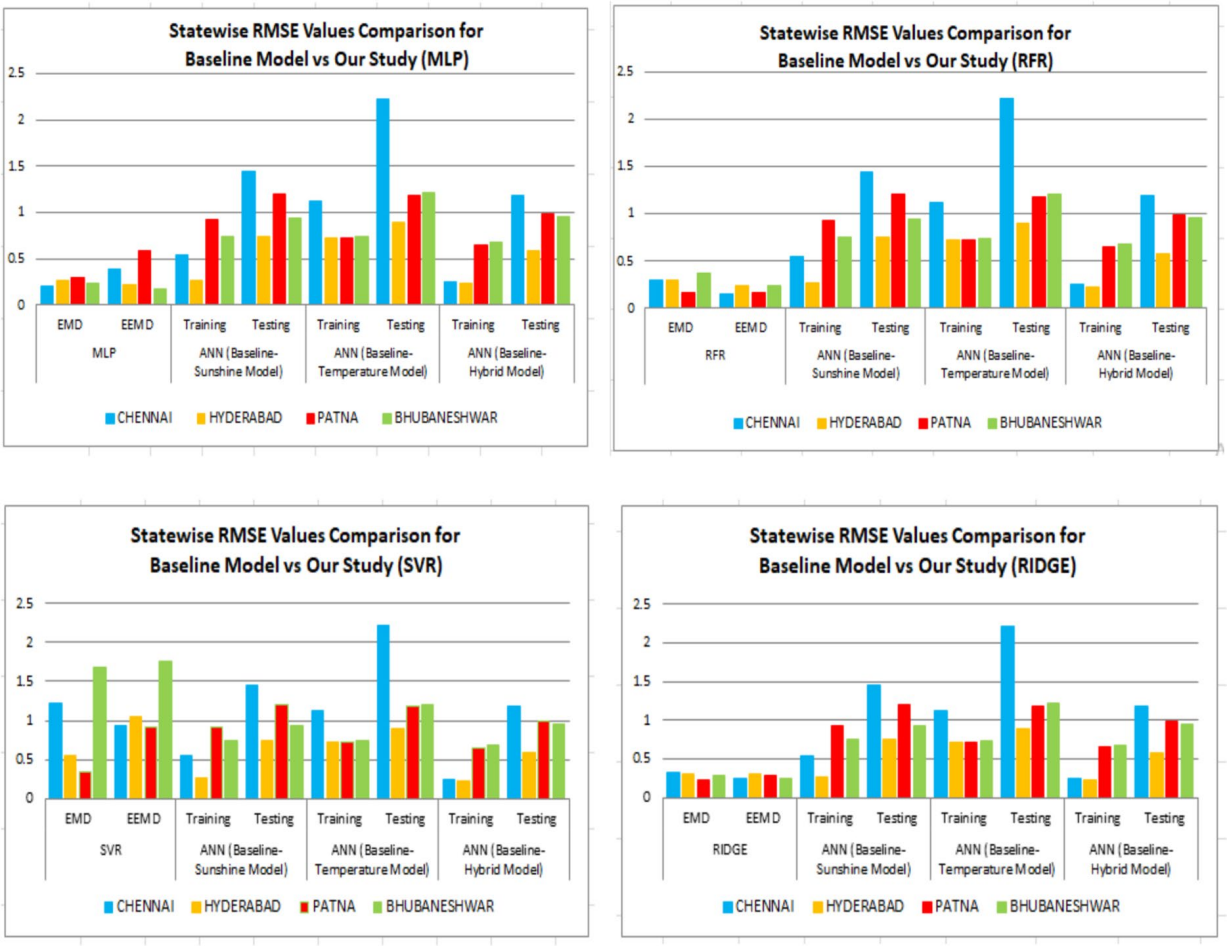
State wise comparison of RMSE values with all algorithms used in our study vs. baseline model for ANN

Figure 30 illustrate the comparison between the values acquired by all the machine learning models used in our study along with the values obtained by the baseline model paper using ANN technique. Results indicate that MLP, RFR, and RIDGE have outperformed the ANN technique while the SVR technique has not performed well in comparison with the ANN technique.

Table 10 compares the MLP-EEMD model employed in this study for monthly solar radiation forecasting with certain other GSR forecasting models found in the literature, with the assessment conducted in terms of RMSE. This is done to ensure that our research is effective. When compared to our newly constructed MLP-EEMD model, the existing prediction models have limited performance, as shown in Table 11.

**Table 11:** Comparison of proposed model with other works

Author/reference	Model	Input parameters	Location	RMSE testing	*R*	*R*^2^
Premalatha and Valan Arasu (2016)	ANN	latitude, longitude, altitude, year, month, mean ambient air temperature, mean station level pressure, mean wind speed, and mean relative humidity	Mumbai, India	3.6461	0.9272	-
Meenal and Selvakumar (2018)	SVM	Tmax(monthly mean maximum temperatures), *T*min (monthly mean minimum temperatures), *S* (monthly mean daily bright sunshine hours), S0 (maximum possible monthly mean daily sunshine hours), H0 (monthly mean daily extraterrestrial radiation on horizontal surface)	Bhubaneswar, India	0.4223	0.9919	-
			Chennai, India	0.8083	0.9465	-
			Hyderabad, India	0.4205	0.9911	-
			Patna, India	0.6515	0.9746	-
	ANN	*T*max (monthly mean maximum temperatures), *T*min (monthly mean minimum temperatures), *S* (monthly mean daily bright sunshine hours), S0 (maximum possible monthly mean daily sunshine hours)	Bhubaneswar, India	0.9377	0.9559	-
				1.2112	0.9334	-
				0.9553	0.952	-
			Chennai, India	1.4468	0.9739	-
				2.2197	0.7727	-
				1.1848	0.9835	-
			Hyderabad, India	0.7451	0.9847	-
				0.8929	0.9725	-
				0.5814	0.9894	-
			Patna, India	1.2003	0.9244	-
				1.1807	0.927	-
				0.9828	0.9465	-
Belaid and Mellit (2016)	SVM	Measured temperature (tmin), extraterrestrial solar radiation (H0)	Algeria	1.524	0.986	-
	MLP			1.596	0.97	-
Anuradha et al. (2021)	RFR	Mean daily values for air temperature, humidity, wind speed and direction, visibility, average values of pressure, wind speed, and electricity generated	India	27.32	-	-
Siami-Namini et al. (2018)	CNN and LSTM	PV module temperature, the current, voltage, frequency, phases, and PV power	China	7.5 min interval:1.30	-	-
				15 min interval:1.40	-	-
				30 min interval:2.04	-	-
Hedar et al. (2021)	GPR	Air temperature, average wind direct at 3 m, average wind speed at 3 m, diffuse horizontal irradiance	China	421.15*r*	-	-
Citakoglu (2015)	Abdalla equation	Average sunshine duration hours (*n*), monthly average daylight hours (*N*), average temperature (*T*mean), average humidity(Rhmean)	Turkey	43.95	-	0.906
	Angstrom equation	Extra-terrestrial radiation (Ra), sunshine hours (*n*) and the maximum monthly mean sunshine hours (*N*)		2.234	-	0.927
	Bahel equation	Daylight hours (*N*), monthly mean sunshine duration (*n*)		2.062	-	0.925
	Hargreaves–Samani equation	Extra-terrestrial radiation (Ra), monthly mean maximum air temperature (*T*max), monthly mean minimum air temperature (*T*min)		18.192	-	0.809
	ANN	Month number (M), extraterrestrial radiation (Ra) average temperature (*T*mean) and average relative humidity (RHmean)		1.65	-	0.93
	ANFIS			1.691		0.926
	MLR			4.745	-	0.81
Dong and Jiang (2019)	CS-hard-ridge-RBF	12 meteorological factors	USA	324.63–417.78	-	-
	DE-hard-ridge-RBF			388.8—472.18	-	-
Olatomiwa (2015)	SVM-polynomial	Monthly mean maximum temperature (*T*max), monthly mean minimum temperature (*T*min) and monthly mean sunshine duration (*n*)	Nigeria	1.510218	-	0.74
	SVR-radial			1.905994	-	0.588
	ANFIS			2.0628		0.547
	ANFIS-ACO			1.5485	-	0.731
	ANFIS-DE			1.601	-	0.693
	ANFIS-GA			1.7696	-	0.635
	ANFIS-PSO			1.8015	-	0.567
Thombare et al. (2022)	ANN	Five inputs	India	1.80193	-	-
		Six inputs		1.68021	-	-
		Seven inputs		1.58994	-	-
Jiang et al. (2020)	ANN	Solar radiation, sunshine hours, rainfall, humidity, temp, ATM (atmospheric pressure)	China	0.746	-	-
Mohammadi et al. (2015)	ANN	Latitude, longitude, altitude, time of day, months, relative humidity, rainfall, air temperature, long wave, length, and wind speed	India	4.5	-	-
Jiang and Dong (2016)	ANN	Latitude, altitude, sunshine percentage, clearness index	China	0.867	-	0.97
Pan et al. (2020)	ANN	Latitude, longitude, altitude	China	0.86	0.89	-
Akarslan et al. (2018)	ANN	Latitude, longitude, altitude, month, mean land surface temperature	Turkey		0.9936	-
Mubiru and Banda (2008)	ANN	Sunshine duration, maximum temperature, cloud cover, latitude, longitude, altitude	Uganda	0.385	0.974	-
Our study	MLP-EEMD	Bright sunshine hours(S), maximum temperature (*T*max), minimum temperature (*T*min)	Bhubaneswar, India	0.18	0.9979	-
			Chennai, India	0.381	0.992	-
			Delhi, India	0.321	0.9969	-
			Hyderabad, India	0.227	0.998	-
			Nagpur, India	0.4156	0.998	-
			Patna, India	0.58	0.98	-
			Trivandrum, India	0.22	0.996	-

## Discussion

In proposed work, the signal processing techniques such as EMD and EEMD with novel ML algorithms for solar radiation forecasting determines significant efficacy in terms of errors RMSE, MAE, and correlation coefficient (*R*). The EEMD method provides a significant improvement in the decomposition effect of the EMD method by reducing modal aliasing. The ML models like support vector regression (SVR), random forest regression (RFR), and RIDGE regression, multi-layer perceptron (MLP) are applied with comparison with baseline models. SVR deals with regression problems, to optimize the continuous valued function, while minimizing the prediction error. RFR is a meta-estimator for regression problems, immune to overfitting and learns spurious correlations of constructed model. Ridge regression eliminates the bias coefficients and reduces the mean square error by shrinking the coefficients of the model in order to reduce the problems of multi-collinearity, overfitting associated with ordinary least squares regression. MLP is a univariate model used for forecasting problems by learning a series of past observations to predict the next value in the sequence. Additionally, several deep learning algorithms, including long short-term memory (LSTM) and bidirectional LSTM (BiLSTM), were applied in conjunction with the proposed decomposition techniques. However, these algorithms exhibited high error rates, with root mean square error (RMSE) values of 0.82 and 0.90 and mean absolute error (MAE) values of 0.60 and 0.62 scores. Furthermore, the computational time required to execute these algorithms was significantly high.

The optimal results are achieved in terms of evaluation criteria such as RMSE, *R*^2^, and MAE. RMSE is a statistical value which assess the largest expected error in the forecasted data. MAE provides the difference between two set of data. A correlation coefficient is a number that measures the strength and direction of a linear relationship between two or more variables. It ranges from − 1 to + 1. For our work, we have mainly considered the article of Meenal and Selvakumar (2018) as our baseline model. Meenal and Selvakumar (2018) have applied the SVM and ANN for the GSR forecast. The four models trained and evaluated for *R* in Table 4, and the diagrammatic representation is shown in Fig. 18. Tables 5, 6, 7, and 8 depict the performance of our study with the baseline model for Bhubaneshwar, Chennai, Hyderabad, and Patna.

Figures 26, 27, 28, and 29 depict the *R* and RMSE values comparison obtained using our study and the baseline model. Table 10 compares the MLP-EEMD model employed in this study for monthly solar radiation forecasting with certain other GSR forecasting models found in the literature, with the assessment conducted in terms of RMSE. This is done to ensure that our research is effective. When compared to our newly constructed EEMD-MLP model, the existing prediction models have limited performance, as shown in Table 11. The comparative analysis of various machine learning models for temperature prediction across different locations in India and other countries reveals significant performance variations. For instance, Premalatha and Valan Arasu (2016) employed an artificial neural network (ANN) model using multiple input parameters, including latitude, longitude, altitude, and various meteorological factors, achieving a root mean square error (RMSE) of 3.6461 and an *R*^2^ value of 0.9272 in Mumbai, India. Conversely, Meenal and Selvakumar (2018) utilized a support vector machine (SVM) model across several Indian cities, demonstrating lower RMSE values, with Bhubaneswar recording an RMSE of 0.4223 and an *R*^2^ of 0.99. Belaid and Mellit (2016) utilized support vector machine (SVM) and multilayer perceptron (MLP) models in Algeria, obtaining RMSE values of 1.524 and 1.596 respectively, with *R*^2^ values of 0.986 and 0.97, indicating strong predictive capabilities. Anuradha et al. (2021) employed random forest regression (RFR) using mean of daily meteorological data, resulting in a significantly higher RMSE of 27.32, highlighting challenges in accuracy when utilizing broader datasets. Additionally, Siami-Namini et al. (2018) explored the application of convolutional neural networks (CNN) and long short-term memory (LSTM) networks for photovoltaic (PV) module temperature forecasting in China, recording RMSE values of 1.30, 1.40, and 2.04 at different time intervals. Furthermore, Hedar et al. (2021) applied Gaussian process regression (GPR) to predict temperature based on various wind and irradiance factors, though no specific performance metrics were provided. In Turkey, Citakoglu (2015) evaluated multiple empirical equations, including the Abdalla, Angstrom, Bahel, and Hargreaves–Samani equations, yielding RMSE values ranging from 2.062 to 43.95, with corresponding *R*^2^ values indicating varying degrees of fit. These findings illustrate the diversity in predictive modeling techniques and the necessity for tailored approaches based on specific environmental variables and data characteristics.

Recent advancements in temperature prediction methodologies highlight the effectiveness of various machine learning models across diverse geographical regions. Dong and Jiang (2019) employed the CS-hard-ridge-RBF model utilizing 12 meteorological factors in the USA, achieving RMSE values ranging from 324.63 to 417.78, while the DE-hard-ridge-RBF model reported values from 388.8 to 472.18, indicating a broad performance spectrum. In Nigeria, Olatomiwa et al. (2015) investigated several models, including a polynomial support vector machine (SVM) yielded an RMSE of 1.5102 and an *R*^2^ of 0.74, while radial support vector regression (SVR) showed a higher RMSE of 1.9059 with an *R*^2^ of 0.588. Additionally, different adaptive neuro-fuzzy inference systems (ANFIS) produced RMSE values between 1.5485 and 2.0628, indicating the impact of algorithm choice on predictive accuracy. Thombare et al. (2022) analyzed the performance of artificial neural networks (ANN) in India, with RMSE values improving from 1.8019 for five inputs to 1.5899 for seven inputs.

Jiang et al. (2020) presented an ANN model for temperature prediction in China with an RMSE of 0.746, integrating factors such as solar radiation and atmospheric pressure. Similarly, Jiang and Dong (2016) and Pan et al. (2020), showed that RMSE values reached 0.997, leading to enhanced accuracy during peak hours and nighttime. Akarslan et al. (2018) further reinforced the robustness of ANN models, achieving an *R*^2^ value of 0.9936, while Mubiru and Banda (2008) reported an RMSE of 0.385 in Uganda, showcasing the versatility and efficacy of these approaches in different climatic contexts. These findings underscore the critical role of model selection and parameter optimization in enhancing predictive performance in meteorological applications. The proposed work, employs a multi-layer perceptron (MLP) model enhanced with ensemble empirical mode decomposition (EEMD), yielded impressive results with RMSE values ranging from 0.18 to 0.58 across various Indian cities, indicating a high predictive accuracy with *R*^2^ values approaching 0.999. These findings underscore the efficacy of machine learning approaches in meteorological predictions, highlighting the importance of model selection and input parameter optimization for enhancing forecast reliability.

However, a significant limitation of the empirical mode decomposition (EMD) algorithm is the phenomenon of mode mixing, where a single intrinsic mode function (IMF) can contain signals of varying scales. Although the ensemble empirical mode decomposition (EEMD) method addresses the mode mixing issue, it results in residual white noise that remains non-negligible after reconstruction, complicating the prediction process. To overcome this limitation, the future work focus on implementing a few signal decomposition techniques such as CEEMD and ICEEMD using deep learning algorithms.

## Conclusion

This paper presents a novel method for forecasting solar radiation in various cities throughout India. The cities included in the study are Delhi, Chennai, Hyderabad, Nagpur, Patna, Trivandrum, and Bhubaneswar. The approach utilized four machine learning algorithms: MLP, SVR, ridge regression, and RFR, along with two signal processing techniques, EMD and EEMD. Among all the models developed, the MLP-EEMD technique yielded the most optimum results in terms of the evaluation metrics. Among all the models built, the MLP-EEMD technique yielded the most optimum results in terms of the evaluation metrics such as RMSE, has achieved 0.381 for Chennai, 0.241 for Delhi, 0.212 for Hyderabad, 0.4156 for Nagpur, 0.58 for Patna, 0.22 for Trivandrum, and 0.18 for Bhubaneshwar. When evaluated against MAE values, our model yielded 0.324 for Chennai, 0.321 for Delhi, 0.227 for Hyderabad, 0.3439 for Nagpur, 0.19 for Patna, 0.202 for Trivandrum and 0.31 for Bhubaneshwar. Finally, when testing for *R* values, the model gave 0.992 for Chennai, 0.9969 for Delhi, 0.998 for Hyderabad, 0.998 for Nagpur, 0.98 for Patna, 0.996 for Trivandrum, and 0.9979 for Bhubaneshwar. When assessed in terms of averages, the model has achieved an RMSE of 0.33 and *R* of 0.994 respectively. In order to further evaluate the efficiency of our proposed work, a comprehensive comparison with 18 prior studies, including a baseline model, revealed that our model had outperformed its potential as reliable, cost effective, and time efficient solution to adapt for the long term solar forecasting. In our study, the machine learning model outperformed the deep learning model due to the relatively small size and nature of our dataset. For future work, we plan to update the dataset by extending it over more years and explore the adoption of deep learning algorithms.

## Data Availability

The data used in this research will be made available upon reasonable request to the authors.

## Declarations

### Declarations

The authors have not used the AI tools to write or generate any content of this manuscript. However, they are used to correct spelling and grammar errors.
